# Condition Monitoring of Rail Transport Systems: A Bibliometric Performance Analysis and Systematic Literature Review

**DOI:** 10.3390/s21144710

**Published:** 2021-07-09

**Authors:** Mariusz Kostrzewski, Rafał Melnik

**Affiliations:** 1 Faculty of Transport, Warsaw University of Technology, 00-662 Warsaw, Poland; 2 Faculty of Computer Science and Food Science, Lomza State University of Applied Sciences, 18-400 Łomża, Poland; rmelnik@pwsip.edu.pl

**Keywords:** condition monitoring, rail transport, rail vehicle, track, monitoring system, sensor, bibliometric analysis, mapping science

## Abstract

Condition monitoring of rail transport systems has become a phenomenon of global interest over the past half a century. The approaches to condition monitoring of various rail transport systems—especially in the context of rail vehicle subsystem and track subsystem monitoring—have been evolving, and have become equally significant and challenging. The evolution of the approaches applied to rail systems’ condition monitoring has followed manual maintenance, through methods connected to the application of sensors, up to the currently discussed methods and techniques focused on the mutual use of automation, data processing, and exchange. The aim of this paper is to provide an essential overview of the academic research on the condition monitoring of rail transport systems. This paper reviews existing literature in order to present an up-to-date, content-based analysis based on a coupled methodology consisting of bibliometric performance analysis and systematic literature review. This combination of literature review approaches allows the authors to focus on the identification of the most influential contributors to the advances in research in the analyzed area of interest, and the most influential and prominent researchers, journals, and papers. These findings have led the authors to specify research trends related to the analyzed area, and additionally identify future research agendas in the investigation from engineering perspectives.

## 1. Introduction

Condition monitoring of certain devices is applied in a particular system in order to detect changes or analyze trends in controlling particular parameters, and most of all to prevent potential failures of a system. Early problem diagnosis ensures preliminary intervention for replacement of any equipment’s components before serious consequences. The term “condition monitoring” evolved—in the case of research connected to rail transport—with the development of instruments that allow the development of increasingly better measurement analyses. Such an evolution of terms, definitions, and approaches connected to condition monitoring in the case of the aforementioned mode of transport is presented in Table 1, based on [1,2,3,4,5,6,7,8,9,10,11,12,13,14,15,16,17,18,19,20,21,22,23,24,25,26,27,28,29,30,31,32,33,34,35,36,37,38,39,40,41,42,43,44,45].

Recent decades have shown a certain evolution of approaches to the condition monitoring of various rail transport systems, especially in the context of rail vehicles and track subsystems. The approaches applied to rail infrastructure condition monitoring have evolved from manual maintenance, through methods connected to the application of sensors, up to the currently discussed techniques focused on the terms of Industry 4.0. The enormous increase in the quantities of generated data—namely, big data—requires diagnostic procedures to be automated. Such a challenge induced the need for up-to-date and improved methods of autonomous interpretation of condition monitoring data (mainly vibration methods). This has led the authors to the aim of this review paper, which is the consideration of several pieces of research focused on the scientific area of rail transport systems’ condition monitoring. The authors of this review paper took into consideration two distinct methods of scientific literature analysis: on the one hand, a bibliometric performance analysis (whose component part is meta-analysis) was carried out, and on the other hand, a systematic literature review was performed.

### 1.1. Background

According to the information contained in key scientific databases, nearly 20 review papers on the condition monitoring of railroad systems have been published in the last 4 decades. This is an insignificant number considering the essence of the subject matter; nevertheless, it is worth underlining that this number concerns some limited areas that will be defined later in the current article. By recalling the published review papers, it can be stated that their authors have taken under consideration topics such as (certainly, the below mentioned topics were considered in research papers as well; however, since review papers are treated as certain summaries, research papers are omitted in the following list):

- condition monitoring techniques for rail vehicle dynamics [10],
- applications and various soft computing methods used for condition monitoring in the context of intelligent systems [46],
- description of onboard condition monitoring systems, methods, and devices—with a particular interest in microelectromechanical sensors, microprocessors, and transceivers—creating wireless sensor networks for freight rail transport [47],
- methods, techniques, and applications connected to the condition monitoring of railroad switches and crossing systems [48],
- condition monitoring systems and enabling automatic railroad track inspection [49],
- railroad track condition monitoring with the use of inertial sensors (inertial measurement sensors) and GPS signals [50],
- brief discussion on machine learning techniques in relation to rail track condition monitoring [51],
- railway turnout condition monitoring [52,53]),
- various aspects connected to the industrial Internet of Things solutions related to condition monitoring [54],
- various types of sensors in wired and wireless sensor networks [23], and wireless-sensor-network-based condition monitoring applied in the case of the rail industry [32,55],
- condition monitoring associated with fault diagnosis of rolling bearings [56].

This briefly presented background allows us to state that no complex review papers connected to condition monitoring for rail transport were noted. As this can be treated as a research gap, the authors decided to consider an in-depth study consisting of a complex review of approaches to condition monitoring for railroad transport, under particular assumptions. This study is related both to passenger and freight railroad transport systems’ condition monitoring—in aspects connected to both rail vehicles and tracks.

### 1.2. The Aim of the Research and Research Questions

As was observed, there is a lack of review papers that treat condition monitoring of railway transport systems as complex systems composed of various facilities and subsystems, straightforwardly discussing both rail tracks and rail vehicles. This review paper’s authors have tried to fill such a research gap. Additionally, we decided to take into consideration the past few decades of condition monitoring considerations presented in numerous publications, which led us to formulate the main research aims of this publication. The research questions are as follows: 

RQ1: What are the current trends in condition-monitoring approaches, and how have these trends evolved over the last decades?

RQ2: What are the future research directions and perspectives in the condition monitoring of rail transport systems?

This paper is organized according to the following structure: The background resulted in research questions that need to be verified based on a certain methodology. The authors present the methodology of the current research in the next section. The two sections following the description of the methodology were selected based on methodological approaches, and are as follows: bibliometric performance analysis, and systematic literature review. Finally, in the last section, we discuss the main results of this study, and additionally present current research limitations and future research agendas.

## 2. Methodology

The type of research methodology and manner of data collection for further analysis depend on the research question being studied. Therefore, before we present our research methodology applied in the case of the current paper, we shall present potential well-known methods and techniques commonly used in the literature review context. Consequently, a proper compilation of methods and techniques is presented at the end of this section.

This paper is based on coupled review methodologies—it combines a bibliometric performance analysis based on content-based analysis, and a systematic literature review as a result of the content-based analysis. The first of these methods is a form of quantitative analysis, while the second is qualitative. As a whole, this methodology adequately presents the full spectrum of publications connected to the condition monitoring of rail transport systems, both in quantitative and qualitative terms.

Neuendorf [57] defined content analysis as a systematic, objective, quantitative examination of a particular message’s characteristics. Various methods and techniques of content analysis are given in Mayring [58]. Content-based analysis is a specific research technique for data feeding applied in order to determine the occurrence of certain words, phrases, keywords, themes, or concepts within some selected, thematically coupled qualitative data. According to Hsieh and Shannon [59], three various approaches to content analysis were verified: conventional, directed, and summative. Working with conventional content analysis starts with observation, and particular codes are obtained based on data analysis. Meanwhile, directed content analysis starts research on a specific theory, and research findings appropriate to the theory that help to obtain initial codes intended for later research, whereas summative content analysis is connected to interpretation of data such as keywords, and further counting and comparisons of keywords or other content. In the case of the latter approach, keywords derive from researchers’ interest or from literature review. Based on such brief definitions, the latter approach was identified as the supporting one for the purposes of this paper. The obtained keywords and elaborated results of content analysis served as a feedstock for further detailed research (i.e., systematic literature review). In the case of research, such sources of data/feedstock can be books, essays, discussions, newspapers, speeches, media, historical documents, etc. In this review paper, these documents are scientific papers, conference papers, books, book chapters, notes, editorials, letters, short surveys, and conference reviews. This is an open catalog of publication types; however, its components are carefully selected in order to answer research questions as precisely as possible.

A bibliometric performance analysis based on content-based analysis is one aspect of the current paper, while the other is the systematic literature review. Literature review in general is characterized by certain typology, which was presented in detail in Marczewska and Kostrzewski [60] based on Grant and Booth, Petticrew and Roberts [61,62]. As was mentioned in Petticrew and Roberts [62], a systematic review’s aim is “to provide an objective, comprehensive summary of the best evidence”. The authors of this paper considered this systematic literature review as a combination of a narrative review (treated as the synthesis of selected research, given in most cases as a descriptive rather than statistical specification Petticrew and Roberts [62]) and a critical review (comparison and evaluation of various perspectives of a particular research problem).

**Figure 1:**
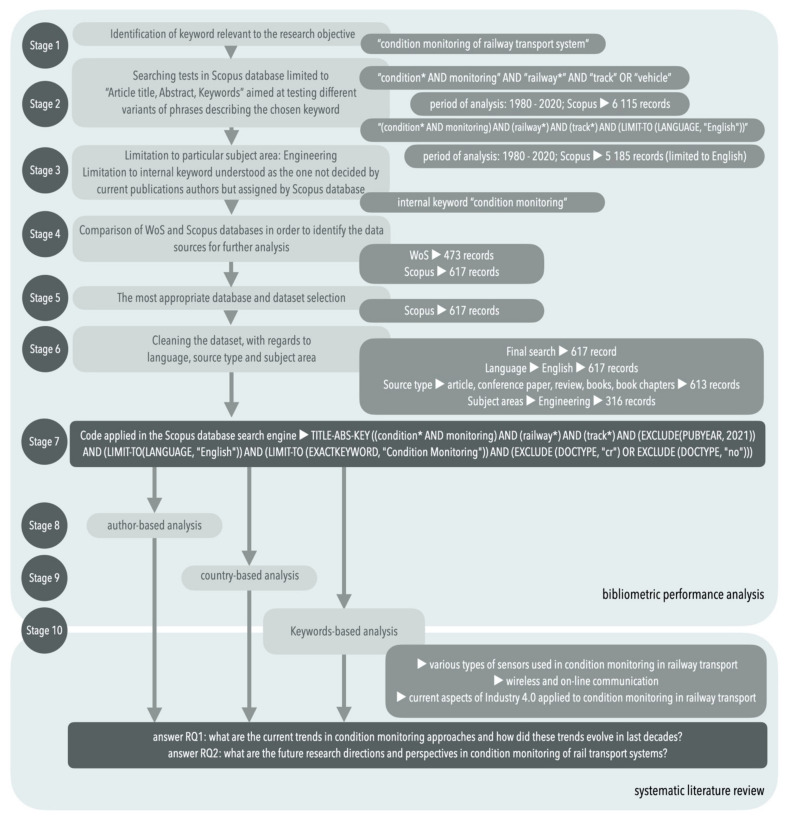
The procedure of the conducted research review. Source: based on [58] (p. 89), [60].

The data searching process was limited to contributions published between 1980 and 2020 (the initiating search was performed on 29 November 2020; however, it must be mentioned that the final search was updated—information about which is discussed in further parts of this paper). The end date was based on the content of the scientific databases. The specific information connected to every stage of the research review is provided in the following subsections. The procedure of this research review is shown in Figure 1.

## 3. Bibliometric Performance Analysis

The first stage of the search process was not limited to any period of time (however, data collection analyses were summarized on 1 July 2020, and then the actualization was obtained on 29 November 2020, and 8 January 2021).

**Figure 2:**
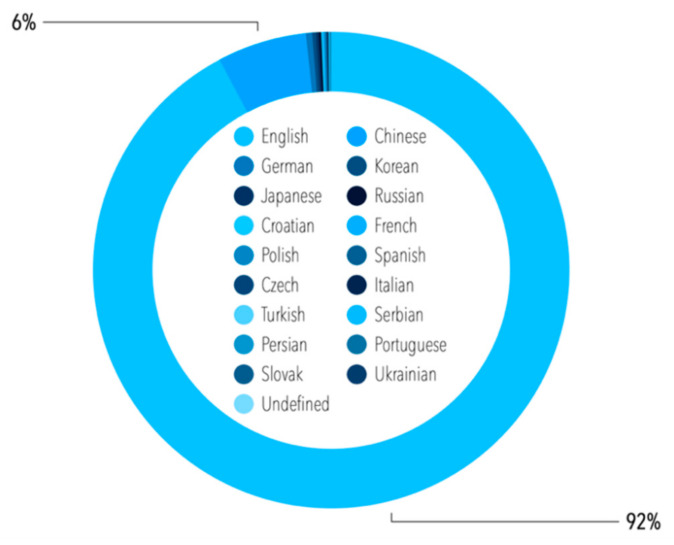
Percentage of publications per language.

Since our interest was focused on the condition monitoring of railway vehicles and tracks, the following set of keywords was investigated: “condition”* AND “monitoring” AND “railway*” AND “track” OR “vehicle”. This resulted in the return of 6115 records in total in the Scopus database (5830 publications before and 285 after actualization). It should be mentioned that 23 publications in total were assigned to the year 2021, and these are included in this number, but excluded from the below statistics. The majority of the publications were released in English, and a significant number of them in Chinese—all of the languages of the publications are given in Figure 2. When the language of the publications was taken into consideration, we decided to analyze only the publications in English (especially given that almost 92% of all of the publications were prepared in English). It was decided to exclude languages other than English, since this language is treated as an international one for the exchange of research and opinions between scientists, and for scholarly communication [63]. The database code “(condition* AND monitoring) AND (railway*) AND (track*) AND (LIMIT-TO (LANGUAGE, “English”))” allowed us to limit the number of searched publications to 5185 items (4946 publications before and 239 after actualization). Once again, it should be mentioned that 23 publications were assigned to the year 2021, and these are included in this number, but not included in the below statistics.

**Figure 3:**
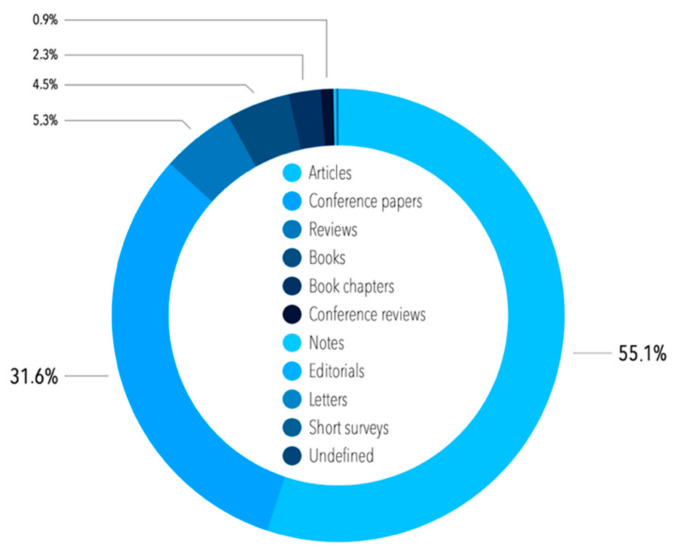
Percentage of publications per document type.

All of the papers in English were also filtered based on their type. As shown in Figure 3, the majority of the publications were scientific articles (55.1%) and conference papers (31.6%).

**Figure 4:**
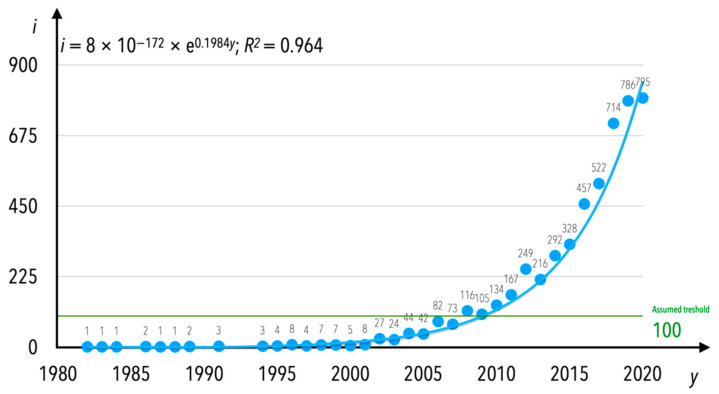
Number of publications (*i*) in the research area of condition monitoring per year (*y*).

The number of publications in the research area of condition monitoring, released in English, grew annually. The number of publications (*i*) in the mentioned research area per year (*y*) is presented in Figure 4. As can be assumed based on Figure 4, the first wave of interest in rail transport condition monitoring was at the end of the 20th century (from the early 1980s). Assuming that 100 publications would be the particular threshold, it can be observed that the second wave of interest was between 2000 and 2007. In the current period of time i.e., 2008–2020, the interest is enormous when numbers are taken into consideration. What might also be observed is that since 1994 there have been no gap years in the publishing of manuscripts in English connected to the analyzed research area. The increase in the number of documents published per year between 1982 and 2020 can be determined by an exponential equation with a coefficient of determination equal to *R*^2^ = 0.964; this equation is noted as Equation (1). The end of the year 2020 was not excluded from this analysis; however, one should bear in mind that the number of publications will increase, considering that indexing in scientific databases is out of regulations; for example, if there was still a month of 2020 left until the end of the period between the completion of the analyzed data and the end of the year, not all of the released publications would have been indexed in the database yet.

(1) $$i=8\times 10^{-172}\times e^{0.1984y}$$

**Figure 5:**
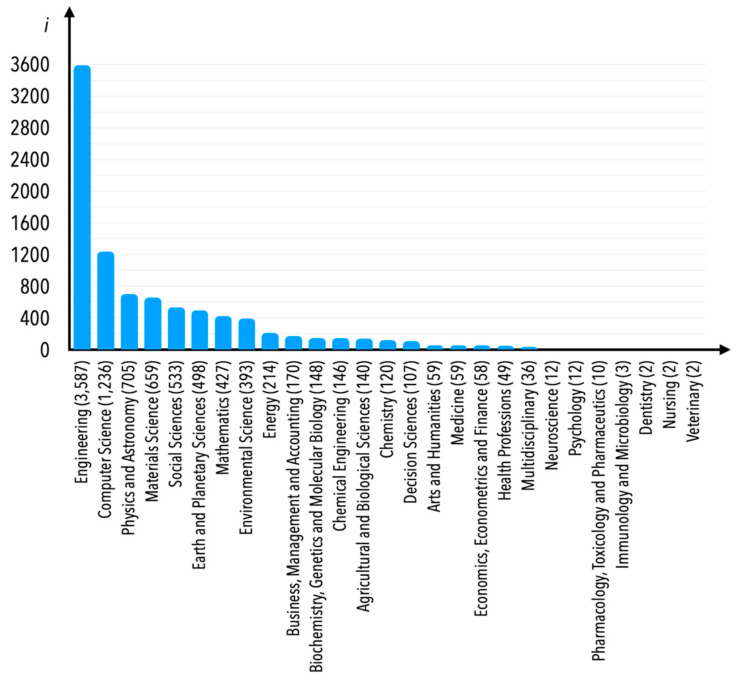
Number of publications (*i*) in the research area of railway transport condition monitoring per particular subject area.

**Figure 6:**
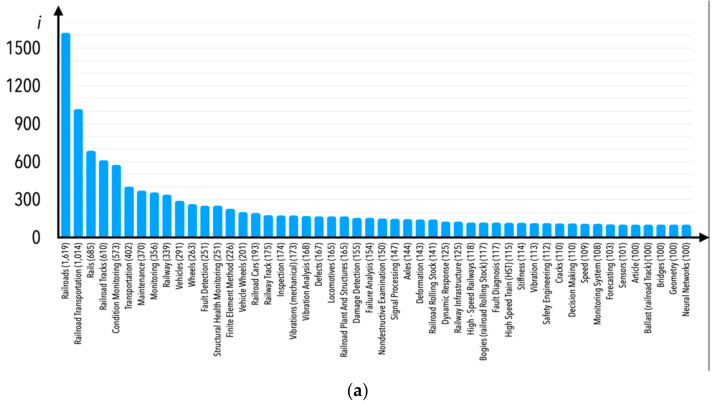

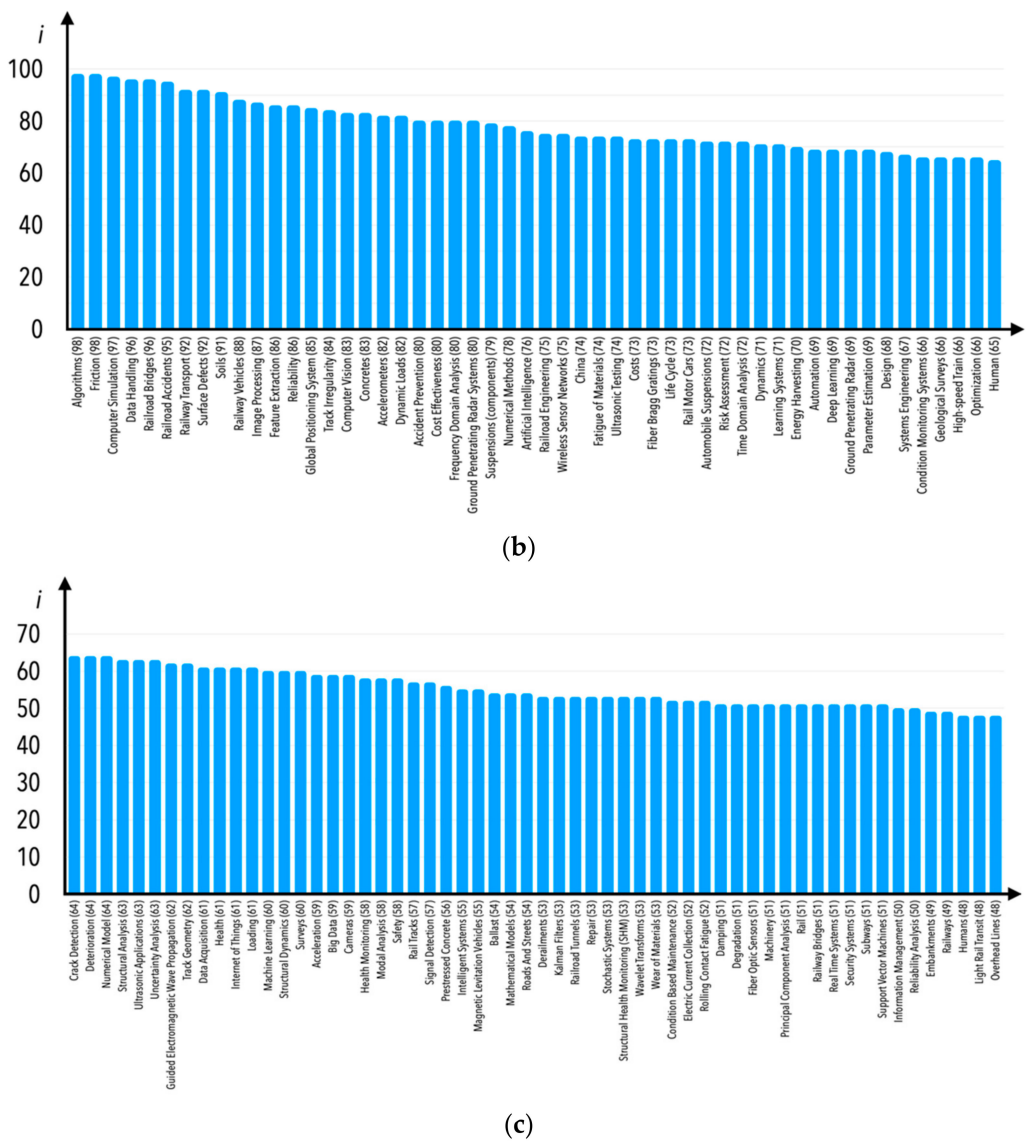
Number of publications (*i*) in the research area of railway transport condition monitoring per particular Scopus internal keywords: a number of publications assigned to internal keywords equal to between 0 and 64 (**c**), between 65 and 99 (**b**), and over 100 (**a**).

In the Scopus database, each paper is classified by subject area. In the case of the analyzed research area, one would consider some of the subject areas mentioned in Figure 5 to be atypical. Nevertheless, we decided not to limit publications according to any subject area; however, Engineering would first and foremost be the area most thematically connected to the subject matter, and secondly the most numerous of all in terms of number of publications. Instead, we decided to limit the publications based on Scopus database internal keywords—internal keywords are understood as those that were not decided by the currently examined publications’ authors, but rather by the employees of the company that owns the Scopus database. These keywords are mentioned in Figure 6a–c (there were 160 Scopus database internal keywords verified; therefore, a figure showing one graph only would be impossible to read in detail—we thus decided to present it in three separate graphs indicating a number of publications assigned to internal keywords equal to between 0 and 64 (part c), between 65 and 99 (part b), and over 100 (part a).

Potential Scopus database internal keywords of interest for the purposes of this paper would be “railroad”, “railroad transportation”, “rails”, “condition monitoring”, “monitoring” (included in “condition monitoring” term), “railway”, “fault detection”, “railway track”, “vibration analysis”, “damage detection”, “failure analysis”, “railway infrastructure”, “fault diagnosis”, “vibration”, “monitoring system”, “railway accidents”, “surface defects”, “track irregularity”, “accident prevention”, “railroad engineering”, “condition monitoring system” (this term is also understood as “condition monitoring”), “track geometry”, “rail track”, “rail”, “railways”, etc. Although internal keywords such as “railroad”, “railroad transportation”, and “rails” describe more publications than “condition monitoring” terms, these are too general, and are characterized by a high degree of abstractness in connection to the particular subject matter. On the other hand, the remaining internal keywords are assigned to fewer publications than “condition monitoring”. Therefore, we assumed that the research publications of interest were hidden in a group focused on the internal keyword “condition monitoring”. Therefore, publications from the circle of this keyword are subjected to analysis in this article.

**Figure 7:**
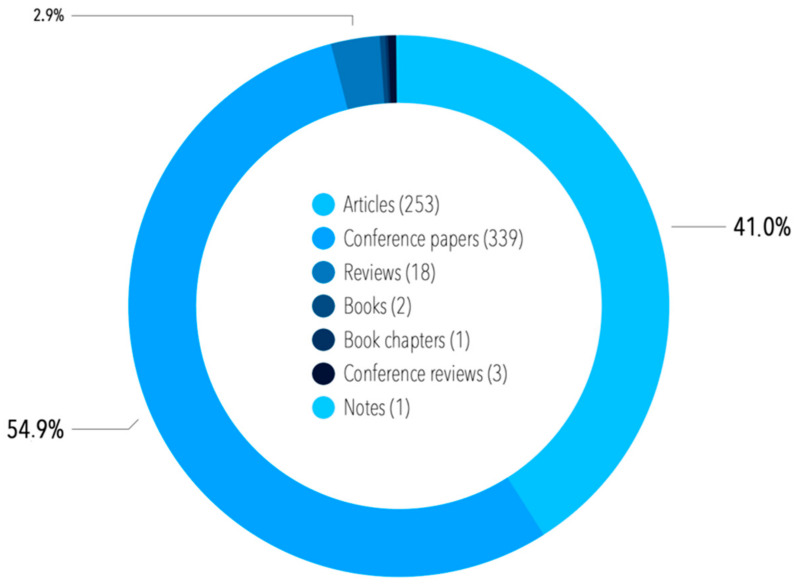
Percentage of publications per document type for the internal keywords connected to condition monitoring.

A total of 617 publications assigned to the condition monitoring term were released in English between 1982 and the first half of 2020 (as mentioned above, some general research of literature was undertaken on 1 July 2020, and then the actualization was obtained on 29 November 2020—since we took into consideration the direct internal keyword “condition monitoring”, the literature review was finally actualized on 8 January 2021, and henceforth, in this publication, the whole period from 1982 to the end of 2020 is assessed). Most of the contributions were classified as articles and conference papers, reviews, and conference reviews; hence, these types of references were of interest to us. Unlike the sample before filtering via internal keywords, more publications in this sample were released as conference papers (54.9%) than articles (41.0%) (Figure 7).

To satisfy the reader’s inquisitiveness, the full code applied in the Scopus database search engine is as follows:

(condition*ANDmonitoring)AND (railway*)AND (track*)AND (EXCLUDE (PUBYEAR,2021))AND (LIMIT-TO (LANGUAGE,“English”))AND (LIMIT-TO (EXACTKEYWORD,“Condition Monitoring”)).

Notes and conference reviews were excluded from the research, since these are believed to be less influential in scientific terms and, thus, less relevant. For this reason, the studies to be assessed were selected from these areas, constituting a final research sample of 613 document results found with the following full code applied in the Scopus database search engine: 

(condition*ANDmonitoring)AND (railway*)AND (track*)AND (EXCLUDE (PUBYEAR,2021))AND (LIMIT-TO (LANGUAGE,“English”))AND (LIMIT-TO (EXACTKEYWORD,“Condition Monitoring”))AND (EXCLUDE (DOCTYPE,“cr”)OR EXCLUDE (DOCTYPE,“no”).

**Figure 8:**
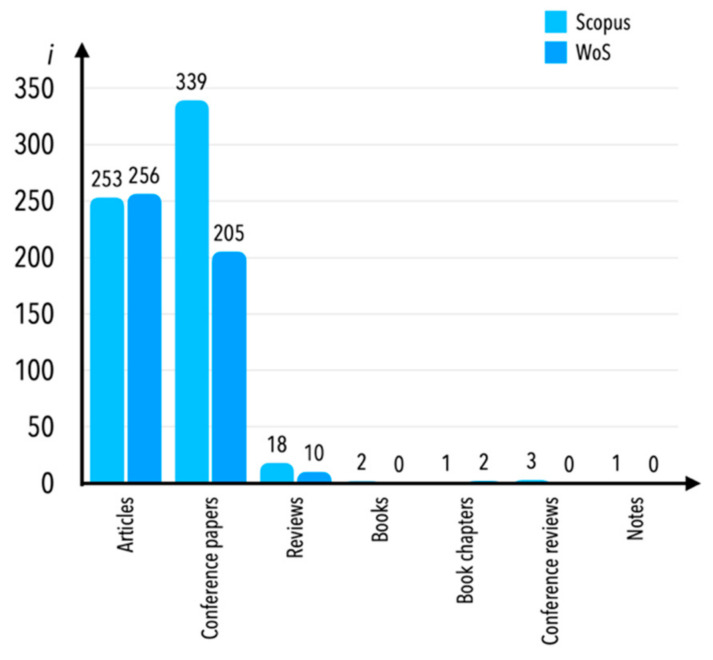
Numbers of publications per document type for the Scopus and WoS databases.

It is worth explaining the choice of the scientific database in these considerations. A couple of years ago, the authors of Baier-Fuentes et al. [64] mentioned that the Web of Science (WoS) database was the primary bibliometric source of scientific evaluation. In recent years, the Scopus database became predominant to WoS. The Scopus database performs similar bibliographic attributes as WoS, such as literature searching and citation analyses of bibliometric records. For confirmation of the validity and adequacy of the investigations, both databases were searched before further analysis. Similarly to searches of the Scopus database, we also found records on the WoS database. Since our interest was focused on the condition monitoring of railway tracks, we investigated the same set keywords on WoS, i.e.,: “condition* AND monitoring” AND “railway*” AND “track” OR “vehicle”. The full code applied in the WoS database search engine was as follows: ((TS=(condition* AND monitoring) AND TS=(railway*) AND TS=(track*)) OR (TI=(condition* AND monitoring) AND TI=(railway*) AND TI=(track*)) OR (AB=(condition* AND monitoring) AND AB=(railway*) AND AB=(track*)) OR (AK=(condition* AND monitoring) AND AK=(railway*) AND AK=(track*))) AND LANGUAGE: (English) AND LANGUAGE: (English) Refined by: DOCUMENT TYPES: (ARTICLE OR REVIEW OR BOOK CHAPTER OR PROCEEDINGS PAPER) Timespan: All years. Indexes: SCI-EXPANDED, SSCI, A&HCI, CPCI-S, CPCI-SSH, BKCI-S, BKCI-SSH, ESCI, CCR-EXPANDED, IC (it should be noted that the search acronyms’ meanings are as follows: AK—authors’ keywords; TS—topic; TI—title; AB—abstract). The search of the WoS database resulted in 473 records (checked on 8 January 2021). The majority of the WoS-indexed publications were also found on the Scopus database. Moreover, the Scopus database is characterized by a higher number of unique journals compared to WoS, as a result of which the total number of records in the Scopus database was greater than in the WoS database. A comparison of the records found in both databases is given in Figure 8 (please note that the type of publications known in Scopus as conferences papers are known in WoS as proceeding papers). Taking all of the above into account, the Scopus database was selected as the main source of bibliometric records for the study presented in this review paper.

**Figure 9:**
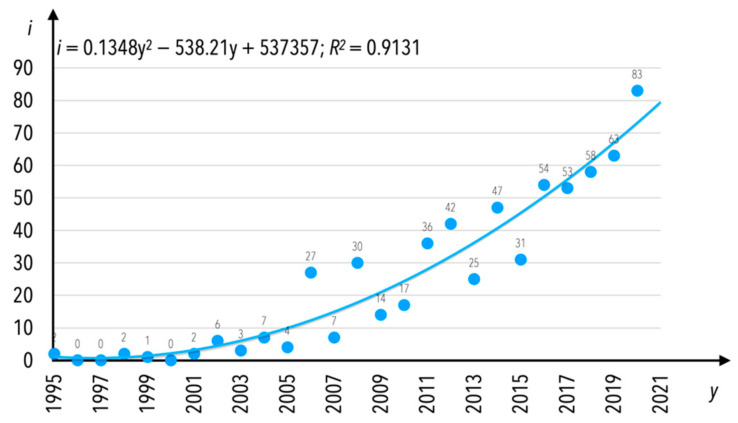
Number of publications (*i*) in the research area of track condition monitoring per year (*y*) for the internal keywords “condition monitoring”.

The increase in the number of publications from one year to another is different when the concept of publications is limited only to those for which the internal keyword “condition monitoring” was indicated. There is no exponential dependence as seen in Figure 3; instead, the increase in the number of publications is characterized by a polynomial trend. A trend line was computed since 1995 (Figure 9); this shows that research continued on a regular basis, but was not “burdened” with the influence of the interest among researchers in certain period-specific terms or keywords. The increase in the number of documents published per year between 1995 and 2020 can be determined by a polynomial equation with a coefficient of determination equal to *R*^2^ = 0.9147; this equation is noted as Equation (2).

(2) $$i=0.1302\times y^{2}+519.86\times y+518956$$

**Figure 10:**
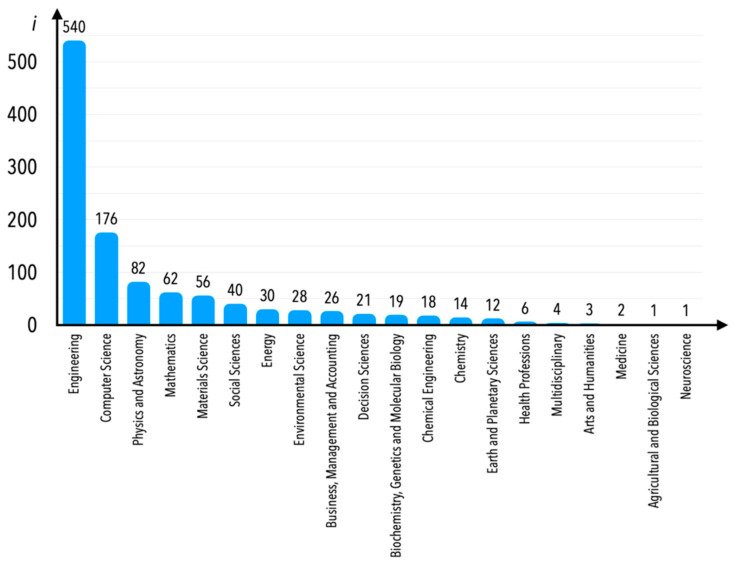
Number of publications (*i*) in the research area of railway transport condition monitoring per particular Scopus research area for the internal keywords “condition monitoring”.

Currently, the particular publication could be assigned to several subject areas, which means that publications assigned to the Engineering subject area can be assigned to several other areas as well. This is based less on the decision of a particular publication’s author(s), but more on an algorithm of the database. As can be observed, in the case of the “condition monitoring” internal keywords, most of the publications are indexed in the Engineering subject area, as is shown in Figure 10. It should be underlined, once again, that this analysis is limited only to publications for which one of the internal keywords was indicated as “condition monitoring”.

**Figure 11:**
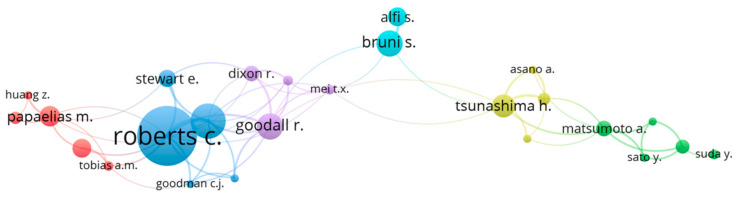
Cooperation in publication and citation of the researchers cited in the case of the internal keywords “condition monitoring”. Source: Own study applied with the use of the VOSviewer software (version 1.6.15).

Cooperation in publication and citation of the researchers cited in the case of the internal keywords “condition monitoring” is one of the most important analyses by which to present the level of researcher productivity. In order to conduct such an analysis, it was important to unify data imported from the database. The set of data connected to the published manuscripts were not given with the unique names or surnames of the selected researchers. Therefore, before analysis, the following unifications were performed: every time “Goodall R.M.” appeared in the data file it was changed to “Goodall R.”; “Weston P.F.” to “Weston P.”; and “García Márquez F.P.” to “Márquez F.P.G.”, etc. After this kind of correction, it was possible to obtain a network of cooperation in publication and citation of researchers cited in the case of the internal keywords “condition monitoring”; This network is presented in Figure 11. To obtain this graph it was assumed that the minimum number of documents indexed in the database per author in the analyzed period had to be equal to five. The minimum number of citations was not limited. 1268 authors were analyzed, and only 57 met the threshold for number of published documents (if we set this threshold to a minimum of 4 documents, the number of authors meeting expectations would be 84; for 3 documents, it would be 144 authors; and for 2 documents it would be 304 authors).

For the total number of 57 unique researchers, the total strength of co-authorship links with other authors was calculated (Table 2; including the list of the researchers’ publications). The researchers with the highest link strength were selected for graphical interpretation, totaling 25 authors.

The larger the diameter of a certain node as presented in Figure 11, the higher the strength of cooperation with other researchers. As can be observed, all of the researchers were divided into six groups (six clusters). It can be noticed that the greatest productivity was that of Roberts C. (the diameter of the node assigned to this researcher shows the greatest productivity in terms of publications), who can be additionally distinguished by having the highest number of co-authorship publications (this researcher was co-author in 34 publications and 748 citations by the time of data collection). Leading positions in other clusters are in the cases of Papaelias M. (12 publications with 605 citations in total), Goodall R. (15 publications associated with the highest number of citations in total, i.e., 814), Bruni S. (15 publications with 284 citations in total), Tsunashima H. (13 publications with 274 citations in total), and Matsumoto A. (9 publications with 48 citations in total).

**Figure 12:**
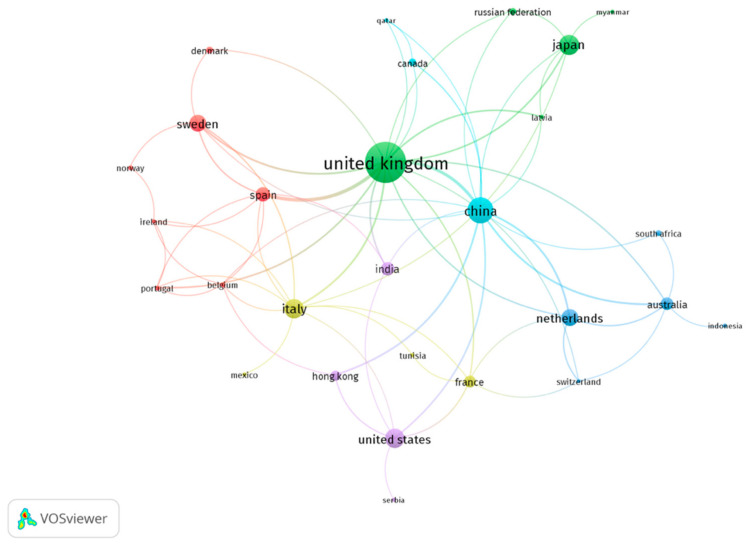
Countries’ specific cooperation in the research on “condition monitoring”. Source: Own study applied using VOSviewer software (version 1.6.15).

Research on condition monitoring is conducted all over the world; however, several countries are characterized with leading roles. The research conducted in the United Kingdom is characterized by the most outstanding number of publications and citations, as is mentioned in Table 3 (moreover, as can be observed in Figure 12, the UK is characterized by the highest strength of interests, as illustrated by its having the highest diameter circle). This is not surprising given the fact the UK is the country of origin of rail transport. The next countries, as sorted by the number of publications, are China, Japan, Italy, the USA, the Netherlands, Sweden, and Spain—or, sorted by the number of citations: Italy, Spain, the USA, China, Japan, and Sweden (the Netherlands, in this case, is placed after several other countries). One can find the reasons for such an occurrence: In most of these countries, high-speed train systems are developed, whose condition monitoring must be treated with the highest interest, for safety reasons. It would be interesting to compare these data with the researchers’ affiliations as well; however, affiliations are not analyzed in this paper. The reason for this is that, according to VOSviewer’s creators, the Scopus database information on organizations may not have been harmonized—that is, organizations may not have a consistent format.

## 4. Systematic Literature Review

**Figure 13:**
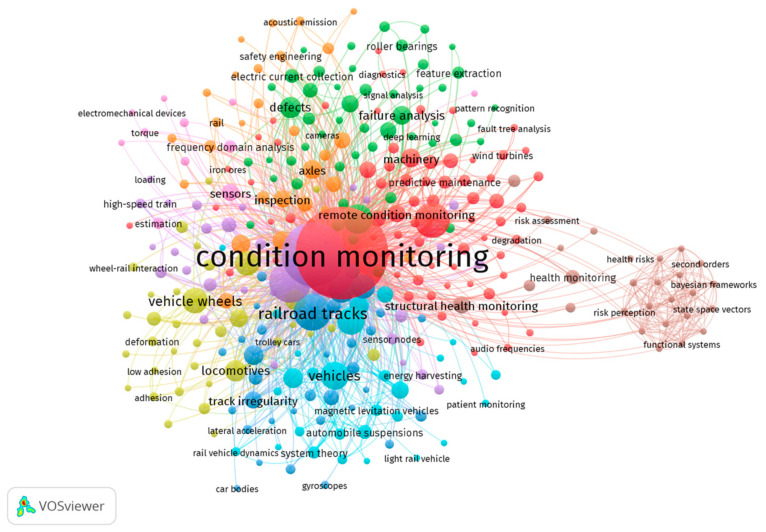
Complete set of keywords significant to the research gathered around the internal keywords “condition monitoring”. Source: Own study applied using VOSviewer software (version 1.6.15).

Keywords are primarily the language constructs that link various publications. Therefore, special attention is given to them in this review paper. All of the keywords for 613 analyzed publications were grouped around the phrase “condition monitoring”. There were 323 individual keywords reported in total. One should bear in mind that some of these are plural and singular forms of the same word (e.g., “accelerometer” and “accelerometers”), which were not unified, as can be observed in Figure 13. From the viewpoint of this review paper, developed especially for a Special Issue of the journal, selected keywords were chosen. All individual keywords were compared with the list of Special Issue keywords and the journal’s aims and scope, according to the best of our knowledge. Keywords directly connected to infrastructure and vehicles, as well as various techniques and methods, were excluded from the graphical analysis. We decided to consider only the keywords connected to the Special Issue of *Sensors*. The list of selected keywords is given in Table 4; it was divided into three separate parts: The first part is connected to various types of sensors used in different solutions applied to condition monitoring in railway transport. The second part presents publications connected to wireless and online communication, which leads directly to the third part, which focuses on current aspects of Industry 4.0 applied to condition monitoring in railway transport. Anytime a keyword given in Table 4 is mentioned, it is henceforth noted in bold in this paper. 

It is worth mentioning, despite perhaps seeming obvious, that different keywords characterize a given publication. Therefore, it is not possible to group all publications characterized by identical keywords in the text within—for example—a single paragraph; this is the purpose of Table 4. It is therefore possible that a publication presented in a given fragment of the current section concerns a different group of keywords. In the content of this section, a given publication is described once, and any reinstated referencing is rare; therefore, within the scope of this description, more than one keyword may occur in the immediate vicinity, which we hope should be a convenience for the reader. It should also be noted that some parts of the paper treat rail vehicles’ and rail tracks’ condition monitoring separately, whereas others do the opposite. We hope that this does not cause undue interference. Moreover, we tried to describe the presented research by maintaining the chronological sequence (naturally, with minor deviations).

### 4.1. Analysis of Various Types of Sensors Used in Different Solutions Connected to Condition Monitoring in Railway Transport

As mentioned at the end of the previous section, we initially present an analysis of selected papers connected to various types of sensors used in different solutions connected to condition monitoring in railway transport The list of review papers’ specific keywords starts with “acceleration sensors” and “accelerometers”. These keywords were ascribed by the authors in both singular and plural form. 

In Liu et al. [198], two measuring systems, namely, Electronic System Analysis Frog—Mobile, and a video gauge, were used for the condition monitoring of railway turnouts. The authors analyzed data measured with a 3D acceleration sensor mounted on the crossing nose in order to obtain data on the three-dimensional dynamic acceleration of the crossing nose and the vertical displacement of the sleeper. The authors analyzed recordings from cameras (installed outside the track) to detect the movements of the targets set on the rail (or sleeper) by analyzing the recorded videos. During a test in the Netherlands, the researchers observed that the vertical acceleration of a railway crossing was dramatically reduced after tamping and, simultaneously, the frequency composition of the dynamic responses also changed. Several years later, the authors Liu and Markine [193] investigated railway crossing degradation. The authors assessed railway crossings with the use of sensor-based crossing instrumentation, and verified obtained measurements with the use of the multibody vehicle–crossing model. The authors’ results showed that the hunting motion (activated by the track geometry misalignment coupled with non-rectilinearity in front of the crossing) of the high wheel–rail impact forces during trains’ passing caused progressive crossing degradation. Adjacent track structures were another reason for crossing degradation, according to the authors’ results.

**Acceleration sensors** were also applied by Känsälä et al. [268]. The authors focused on the monitoring of rail track condition in Finland with use of sensor technology employing wireless three-dimensional acceleration (as can be observed, various research teams use similar keywords; however, with the different description, as for the previously mentioned paper, it was “3D acceleration sensor”). The system was installed onboard. Anomalies during rides were compared to known, previously measured parameters, for example on bridges and switches. The authors applied map-matching and Bayesian filtering, with the aim of improving the global positioning system (GPS) location accuracy of the measured and collected data. The authors claimed that measurements collected by sensors attached to rolling stock support strategic and operative maintenance planning.

The next keyword connected to this subsection, namely “accelerometer(s)”, was found in the case of the following publications. 

The system described in Danneskiold-Samsøe and Ramkow-Pedersen [269] consisted of an array of **accelerometers** that were applied in order to measure the wheel profile wear. Additionally, this was measured with laser scanners and cameras a manufacturer other than that of the system of accelerometers. Such a combination of devices ensured diagnosis of defects of various types, such as wheel flats, out-of-round wheels, and corrugated wheels.

In Weston et al. [156], the authors observed selected features in the bogie and axle box obtained using robust sensors. Sensors such as accelerometers and rate gyroscopes were mounted on the in-service vehicle’s bogie and axle boxes. The authors claimed incompleteness of the track geometry information obtained via these sensors. In Weston et al. [156], the authors applied a pitch-rate gyroscope mounted of a bogie in order to observe mean vertical track geometry, as well as a bogie-mounted yaw-rate gyroscope to observe mean lateral alignment and wavelength track curvature. The authors described the results of measurements carried out on two separate railway vehicles as a means of research validation. Aside from mean vertical and lateral alignment irregularities, the measurements were also connected to cross-level and twist faults. The authors of Weston et al. [153] mentioned that the optical sensors that were used in rail track condition monitoring systems in the years of the authors’ research were a challenging part of the systems, since these kinds of equipment need frequent cleaning. The authors also added that a simpler and more cost-effective alternative could be using a small number of accelerometers and rate gyroscopes. The results of their research proved that accelerometers and displacement transducers can provide more accurate results. At the same time, a pitch-rate gyroscope method was acclaimed as providing results over a wider speed range, and the use of gyroscopes was also supposed to be a simpler and more robust arrangement. Vertical track irregularities were considered in Weston et al. [153], whereas lateral ones were presented in Weston et al. [154]. In the latter paper, the authors presented measurements (lateral acceleration or yaw rate) with sensors mounted on a bogie for estimation of the mean track alignment, with no application of optical or contact sensors. The results showed that a bogie-mounted yaw-rate gyroscope can provide measurements sufficient to specify maintenance operations laterally; therefore, a sensing accelerometer is not required to monitor the lateral motion of a bogie in response to track alignment (however, the authors claimed this was not straightforward).

In Lee et al. [270], accelerometers were fitted on high-speed trains’ axle boxes and bogies. Measurements of acceleration in both the lateral and vertical directions were compared to a commercial track geometry measurement system, which enabled authors to obtain the following conclusion: The measurements obtained from bogie-mounted accelerometers provided more reliable results than measurements from the axle-box-mounted accelerometers in the waveband from 70 to 150 m. This was unexpected, as the author mentioned, because axle boxes follow tracks’ irregularities more closely than a bogie as a general rule. This research was announced in Lee et al. [271], where a track geometry measurement system equipped with a laser, a camera, and accelerometers was also used to obtain commensurate measurements for high-speed trains.

The authors of Chellaswamy et al. [272] investigated fuzzy logic for the estimation of potential irregularities in the railway track during train operation. The authors elaborated a fuzzy track monitoring system, whose essential parts were **vibration sensors** mounted on an axle box and a car bogie, for measuring acceleration in both the lateral and vertical directions. The system was equipped to automatically track the location of vibrations and send the information to the central office; when the GSM signal was inaccessible, the fuzzy track monitoring system automatically stored the coordinates. The authors concluded that for vertical track irregularity, an accelerometer placed on a bogie gives better results than one on an axle box in a speed range from 51 to 100 km/h. Therefore, their results were consistent with Lee et al. [270]. Chellaswamy et al. [273] described a system—namely, an intelligent track monitoring system—that can be adopted to eliminate the track irregularities. The authors developed MEMS technology and placed **sensors** on both an axle box and a bogie—the measurements were obtained in both the vertical and lateral directions. A global positioning system (GPS) was applied to determine the location within the system; however, if the signal was missing then an intelligent track monitoring system automatically stored the coordinates. Similarly to Chellaswamy et al. [272], the authors stated that a vertical track irregularity MEMS mounted on a bogie gave better results than a MEMS mounted on an axle box for a speed range from 51 to 100 km.

In Bagshawe [274], the author compared servo **accelerometers** with the accelerometers used in MEMSs for track condition monitoring, with a bogie-mounted installation. The author selected a vertical profile as the subject of a comparative trial between the alternatives. According to the presented results, the MEMS accelerometer offered comparable performance at speeds above 50 mph (80.47 km/h), and it was especially a matter of interest that solutions with the use of MEMSs are typically less expensive; **gyroscopes** were also included in the system.

Vertical and lateral acceleration (with use of three accelerometer signals) of the axle box, bogie, and car body were also the sources of measurements in Qin et al. [275]; as in the case of several aforementioned research elaborations, this paper focused on the design of an onboard device for track fault diagnosis and rail irregularity data collection. The authors proposed a system for the calculation of track fault probabilities. 

In Yeo et al. [242], the condition of the track geometry was assessed with the use of a system that consisted of three **accelerometers**, three **gyroscopes**, a tachometer signal, and a GPS feeder comprising an inertial measurement unit; this system was mounted on the bogie. The aim of the authors was to investigate the renewal, degradation, and maintenance of rail tracks.

The system presented in Tsunashima et al. [91] consisted of **accelerometers**, a rate **gyroscope**, a GPS, and a map-matching algorithm applied in order to pinpoint the location of faults on the tracks. The system allowed the authors to estimate track irregularities from accelerations in the vertical and lateral directions, and also to estimate the roll rate of a car body. In order to collect the data for track maintenance, the system was equipped with a smartphone to send the data to a server. Track irregularities for conventional railways were also estimated based on acceleration measurements in the vertical and lateral directions and the roll rate of a car body in Tsunashima et al. [92]; this system’s device was developed to give higher performance for practical use; therefore, the compact onboard devices with **GPS** systems were fitted with a **map-matching algorithm,** as in Tsunashima et al. [91] and Tsunashima et al. [204]. The authors mentioned that their system provides the possibility of the detection of rail corrugation from cabin noise via the computation of spectral peaks. Additionally, a smartphone was proposed as equipment to send data.

Tsunashima et al. [224] developed a system allowing for the estimation of the track geometry and irregularities of Shinkansen tracks using car body motions alone (A Kalman filter was applied to solve the problem of not using other **accelerometer** placements). Their aim was to examine track tamping, with a view to analyzing acceptable quality of riding comfort. The authors claimed that vertical irregularity estimation of tracks is possible, with acceptable accuracy for actual use. 

Continuing description of the works under Japanese supervision, the authors of Tsunashima et al. [89] and Tsunashima et al. [90] presented an onboard sensing device equipped with sensors and a **GPS** system, and diagnosis software that detects track faults based on the root mean square of acceleration obtained from measurements in a car body. Estimation of track irregularity based on car body acceleration was applied with the use of a Kalman filter.

In Xu et al. [276], the authors presented a fault identification method for determining the dynamic management levels of track irregularity based on an evidential reasoning rule (a classification method using a novel combination rule as in Xu et al. [277]); in their research, the authors used sample data measured from two **accelerometers** mounted on an axle box and a car body of an in-service train. 

Milne et al. [278] focused their research on the issue of cumulative distribution functions, and their utility to identify the at-rest position in track deflection data obtained from geophones (for velocity measurements) or **accelerometers**.

Tešić et al. [279] developed software that helps to discover and interpret typical situations connected to irregularities on railway tracks based on measurements from five **accelerometers** (located on the track, body, and axles). Chellaswamy et al. [280] developed a differential evolution algorithm in order to optimize the values of irregularities that were received from bogies’ and car bodies’ acceleration measurements by **MEMS sensors** (accelerometers). These measurements gave the authors actual data about track alignment, which was further investigated with use of the mathematical model and the frequency response analysis.

In Somaschini et al. [281], the authors took into consideration the condition monitoring of insulated rail joints, which to date were monitored directly by trained personnel. However, in-person control of a railway line is time-consuming, and no objective parameters can guarantee the health condition of a joint. Therefore, the authors developed a system consisting of **accelerometers** placed on two wheelsets of a rail vehicle.

Monitoring of the track condition for light rail transport (trams) was also of interest to researchers. In Vinkó and Bocz [282], the authors described a system whose purpose is to record the data of triaxial wireless accelerometers attached to the wheel discs of a regular, in-service tram. The authors mentioned that sensors mounted on axle boxes and bogie side-frames are commonly used worldwide to identify track irregularities. At the same time, they proved that wheel-disc-mounted sensors provided a more adequate solution for monitoring light rail transport tracks. On the other hand, metro track maintenance was, to date, investigated by in-person inspection, according to Komalan and Chauhan [283]; the authors developed a **wireless sensor network** (**WSN**) with sensor nodes including accelerometers laid along the tracks’ length; in their opinion, maintenance time can be reduced thanks to measurements obtained with the application of such a network. This reduction could be ensured through automated monitoring by detecting faults before escalation.

Aside from using accelerometers for track condition monitoring, some significant elements of rail vehicles are also of interest to researchers.

A system for heavy-haul wagons was proposed in Li et al. [110] in order to bolster spring fault condition monitoring; the authors designed the system relying solely on two **accelerometers** (excluding other measuring appliances) mounted on the front left and right rear part of each car body on a heavy-haul train. 

Hajovsky et al. [284] developed a less typical condition monitoring system; the authors’ concept focused on the implementation of a protective fence installed above railway tracks. Such a fence ensures safety against eroding rocks and other material falling from hillsides next to railway tracks. These monitoring systems consisted of fiber optics, as described in Guo et al. [285], and the system was equipped with **accelerometers** (**MEMS sensors**), whereas the energy for the system came from solar panels, as such a system can be installed more easily in hard-to-reach terrain.

Since other significant elements are mentioned, it is worth adding another keyword here—namely, “acoustic emission sensors”—which was taken under consideration in the following publications: [165,166,168,286].

In [166], the authors researched high-frequency acoustic emission pairwise vibration analysis of measuring devices installed on a train in order to investigate axle bearing defects and potential wheel flats. Acoustic emission sensors coupled with accelerometers were installed on an axle bearing box, with minimum intervention within the vehicle’s equipment, as required. 

The authors of Papaelias et al. and Huang et al. [165,168] stated that hotbox wayside systems are subjected to false alarms, and faulty axle bearings can only be detected shortly after failure and fast enough to prevent their effects. Therefore, the authors developed an acoustic emission system (with **acoustic emission sensors** included) for the detection of axle bearing faults. Such a solution was based on the physical dependence on the acoustic emission signal, which arises from the axle bearing fault, resulting in noise and interference (ultrasound). The tests were conducted on actual freight wagons with artificially damaged axle bearings. One paper Fararooy and Allan [286] was related to the condition monitoring and diagnostics of railway signaling devices; the authors employed acoustic emission and other types of sensors, with the application of techniques employing neural networks.

Bearings used in freight service are subjected to rolling contact fatigue. According to Tarawneh et al. [287], subsurface inclusions in bearings resulting from steel contamination initiate subsurface fatigue cracks, which propagate continuously. Therefore, the authors described prognostic models for spalls initiating on bearings’ inner (cone) and outer (cup) rings, which together with a harmonious, **sensor**-based, bearing-condition-monitoring algorithm can increase safety in the use of bearings.

In Sánchez et al. [288], the authors applied two separate approaches in order to detect faults in railway axles. The first of the approaches was connected to gathering and analysis of acceleration signals measured in the longitudinal direction. Meanwhile, the authors applied the data fusion technique in the second approach, which led to evaluation based on measurements obtained with the use of six **accelerometers** by merging the indicator conditions according to the sensor placement. The total number of condition-monitoring indicators per vibration signal was equal to 54. To find the best features, the authors used the mean decrease accuracy method of random forests. The results showed that a fusion of approaches and, consequently, indicators worked efficiently together in order to detect a crack fault in the analyzed rail vehicles’ parts.

Condition monitoring concerns various rail vehicles, passenger and freight cars, and rail infrastructure—especially tracks. Some studies were also conducted in order to monitor the condition of rail bridges, as in Faulknet et al. [263]; the authors developed a rotational measurement system, which operates by measuring quasistatic and dynamic measurements of a bridge, and which consists of **accelerometer(s)** and **gyroscopes**. Gyroscopes were treated in this system as complementary sensors for the instrumentation of the rotational accelerometer. The authors used sensor fusion techniques to combine the measurements, which resulted in analysis of a bridge’s tilting behavior.

Some studies were focused on freight rail transport only (the presented solutions are connected to cars; nevertheless, energy harnessing may also be applied for sensors analyzing tracks; therefore, it is also included in this review paper). The authors of Gao et al. [142] noted that electrical wires are not provided for freight wagons; therefore, power monitoring sensors such as pressure sensors and **accelerometers** cannot be directly applied for such vehicles’ condition monitoring. Nevertheless, these types of sensors are essential for ensuring operational safety; therefore, the authors investigated the approach of harnessing freight wagons’ vibrational energy in order to power monitoring sensors (a pressure sensor and a triaxial MEMS accelerometer) with the use of an electromagnetic vibrational energy harvester with an inertial pendulum. The development of rail transport evoked a dramatic increase in the market requirement for rail-side monitoring devices and sensors in China, as mentioned in Gao et al. [146]; the authors developed a prototype for the smart monitoring of underground rail transit; the power supply would be provided by local energy generation; as a local energy source, the authors considered an electromagnetic energy harvester. Power for the sensor nodes of a wireless sensor network (WSN) was obtained by connecting an accelerometer, a wind pressure sensor, and a temperature sensor. The mentioned sensors were coordinated by a self-powered ZigBee wireless sensor node, which was also developed in Gao et al. [145] for other purposes, mentioned further in this section.

Among the publications listed in Table 4, next to the keyword “accelerometers” are [289] by Farkas—whose authors reviewed measurement of railway track geometry—and Chia et al. [50], in which the authors reviewed application considerations, current challenges, and prospective opportunities for improvements in railroad track condition monitoring with the use of **inertial sensors** (**inertial measurement sensors**) and **GPS** signals. Muthukumar and Nallathambi [49] very briefly reviewed selected condition monitoring systems and algorithms connected to them—namely, automatic railroad track inspection, and **remote sensor networks** based on fuzzy logic. In Yella et al. [290], the authors’ review paper was connected to **artificial intelligence**. Another review paper’s author described detailed information on onboard condition monitoring devices, with a particular interest in treating them as **sensor nodes**—namely, microelectromechanical sensors, microprocessors, and transceivers—creating **wireless sensor networks** Bernal et al. [47]; the authors also investigated systems, methods, and techniques, focusing on solutions connected to applications for freight wagons without onboard electric power. They also mentioned, as in Fraga-Lamos et al. [54], that such systems connected to the Internet of Things network enable advanced data analytics. The authors of Hamadache et al. [48] reviewed methods, techniques, and applications connected to the condition monitoring of railroad switches and crossing systems.

The following keywords were assigned only once: **angular rate sensor**, contrast sensor, eddy current sensor, and infrared proximity sensor. The publications in which each keyword was assigned are mentioned below.

The authors of Sakamoto et al. [259] developed a system consisting of an angular rate sensor mounted on a bogie in order to monitor and detect flange climb derailment that occurs for a railway vehicle at low speeds. 

In Liu et al. [291], a high-speed frame-grabber system was designed in order to monitor bullet train tracks with the use of image recognition. The system included a fast camera, an image acquisition card, artificial lighting, a **contrast sensor**, and other equipment. Results of this research showed that the height of camera installation did not affect the accuracy of the designed system.

The rail fastening, which fixes the rails to the sleepers, is one of the crucial components in rail tracks. Its significant function is connected to maintaining the track gauge and stability. In Chandran et al. [233], the authors took into consideration the monitoring of rail fastening conditions with the use of an **eddy current sensor** system for fastener detection. This kind of sensor applies the principle of electromagnetic induction. The system proposed by the authors was able to detect an individual rail fastening from a height of 65 mm above the rail.

The authors of Kuutti et al. [292] presented their research on the energy efficiency of two escalators at different metro stations. **Infrared-proximity-sensor**-based pedestrian counting can be used in the condition monitoring of escalators, according to the authors. The authors also mentioned that the adjustment of escalators’ energy-saving settings can be analyzed based on periodical pedestrian patterns (power consumption was also analyzed).

The electric sensing device is the next keyword that focuses the interest of a group of researchers.

One paper by Zhang et al. [293] brought attention to the increasing number of **sensor nodes** creating **sensor networks**; the authors observed that the tracking and allocation of fiber Bragg sensors in **electric sensing devices** was becoming a bottleneck in condition monitoring; however, their research was not directly connected to rail transport.

The authors of Zhang et al. [294] developed a system consisting of a fiber Bragg **sensor network** (consisting of **fiber-optic sensors**, also known as **optical fibers**) dedicated to track condition monitoring, especially for high-speed train systems. This condition-monitoring system (with an **electric sensing device**) enables measurements of railway track temperature, displacement, and strain. The authors’ aim was to show that the system was adapted to the complex natural environmental terrain around railways. 

In Anastasopoulos et al. [295], the authors investigated the ability to obtain sub-microstrain accuracy with fiber-optic Bragg gratings, using the authors’ then-novel optical signal processing algorithm designed for the identification of wavelength shifts with high accuracy and precision. The paper was not directly related to rail transport; however, it mentions that **fiber-optic** sensors had been used for the real-time monitoring of railway infrastructure. In Roveri et al. [296], the authors suggested a fiber Bragg sensor array system (**electric sensing device**) mounted along a railway track, for various purposes; on the one hand, the authors planned the monitoring of underground railway traffic (such as the number of axles, the train speed, and load) in real-time, and on the other hand, their aim was the condition monitoring of both the railway track and the train wheels, such as the estimation of rail and wheel wear.

Meanwhile, in relation to the previous keyword, Song and Sun [297] analyzed wheel defects. The authors researched wheel polygons in particular, with a system based on **piezoelectric sensors** (electric sensing devices) instead of strain gauges, which has the advantages of high sensitivity, wide frequency response, vast dynamic range, and perfect electromagnetic-immune properties.

A fiber-optic sensor, mentioned previously by Zhang et al. (2017a) [294], is the next set of keywords worth consideration.

According to Ilie and Stancalie [298], many rail systems consist of **optical fiber** communications infrastructure, especially in urban areas, and this is why the authors mentioned that it is possible to apply optical fiber sensors for the structural health monitoring of railways. In Ilie and Stancalie [298], the authors decided to analyze rail infrastructure in order to avoid broken rails, which might have serious implications for safety in rail transport. The authors’ investigations referred to the maintenance of **fiber-optic sensors**, and the response of this type of device to temperature and strain changes, in order to evaluate railway expansion. In this particular paper, a tested metal probe—similar to the rail material—was exposed to changes in temperature.

Naderi and Mirabadi [299] mentioned the limitations and capabilities of various sensors in railway transport, which resulted in the choosing of a suitable **fiber-optic sensor** for detecting several different parameters. The authors proposed a sensor for the purpose of measuring the train’s weight in different conditions. In Elia et al. [103], the authors compared **fiber-optics** measurement with signals from accelerometers.

In Buggy et al. [300], the authors presented their preliminary research on the dynamic load monitoring of fishplates, stretcher bars, and switchblades on a tram network with the use of optical fiber Bragg grating sensors (also referred to as fiber-optic sensors, **optical fiber Bragg grating strain sensors**, or strain sensors). Arrays of **fiber Bragg grating sensors** were also studied in Buggy et al. [301] for the dynamic load monitoring of fishplates, switchblades, and stretcher bars on a tram network. According to the authors, the measurement of strain on a fishplate can provide information on the torque of the bolts. Optical **fiber Bragg grating sensors** were applied for monitoring changes in the dynamic strain signature that appeared in the abovementioned track elements in response to the passage of a tram over the sensor location, as presented in Buggy et al. [302].

Freight rail airbrakes might affect train scheduling in a negative way, which is why the authors of Poddar and Lipsett [303] decided to research condition-monitoring techniques for airbrake leakage detection. The authors worked on qualitative and quantitative evaluation and verification of the implications of detecting leaks ultrasonically in cold-weather conditions (according to the Scopus index keywords this paper was connected to **fiber-optic sensors**). 

Ni and Zhang [304] suggested approaches in Bayesian modeling for the evaluation of wheel quality. They applied this modeling to a track-side monitoring system based on **optical fiber sensors** in order to obtain data for rail bending and the condition of wheels.

An optical fiber sensing network was also depicted in Tam et al. [305]; the authors developed a condition-monitoring system based on machine learning in order to detect and identify various types of track defects (rail corrugations, dipped weld joints, and defects of rail crossings).

Facilities other than bogies and tracks in the railway vehicle train need condition monitoring as well. Lin et al. [306] analyzed railway bridges; the authors assessed a skewed half-through plate girder railway bridge with the use of a **fiber-optic-sensor**-based (**fiber optics**) monitoring system installed directly on a bridge during its construction. The authors analyzed and evaluated the impact of axle load distribution through the evaluation of track ballast specially allocated along the bridge structure. Meanwhile, the authors of Sengsri and Kaewunruen [307] reviewed the publications connected to bridge bearings as among the most common causes of bridge failure. The review was connected to the use of **sensors** and other such devices.

In Na et al. [308], the authors examined the contact condition of the energy supply system. This contact was certainly associated with a pantograph and an overhead contact line, and the authors analyzed it via image processing implemented using a device installed on a rail vehicle’s roof; this device included **fiber-optic sensors**. In Arthington et al. [309], the authors described their image processing approach; they suggested a technique by which to extract the vertical movement of a pantograph from images taken using existing onboard cameras. Based on the post-processing of images, the contact force between the pantograph and the overhead line can be estimated based on the observed acceleration of the pantograph head. In this solution, Kalman filters are provided to eliminate measurement errors and sensor noise.

Xu et al. [190] studied prestressed concrete sleepers enriched with **fiber-optic-sensor**–based systems, which were called self-sensing sleepers. The authors conducted laboratory tests in order to develop an estimation of rail seat load, detection of cracking, and identification of ballast settlement—all with the use of self-sensing sleepers. The authors concluded that a self-sensing sleeper may be deployed on an operational railway in order to provide reliable and long-term measurements of rail axle load and ballast pressure. Measurements obtained in laboratory tests and based on the FEM simulations showed that this kind of railway element can be used as a rail seat load sensor and a ballast pressure sensor in actual application on tracks.

Optical fiber Bragg grating strain sensors are thematically connected to fiber-optic sensors; therefore, some publications’ descriptions are given directly in the following few paragraphs (with disregard for any alphabetical list of keywords).

In Ho [160], the author used photonic sensors with **fiber Bragg grating** arrays in order to measure the temperature and strain applied in various railway transport solutions. The same year, the research team in Ho et al. [161] presented a solution that applied fiber Bragg grating sensor arrays for train detection, train weight measurement, and data collection for characteristics of wheel–rail interaction in order to enable rail defect detection.

In Wei et al. [37], the authors described a real-time system that was designed to monitor wheel defects based on **fiber Bragg sensors**. This system ensures the minimization of the risk of wheel deterioration by monitoring typical train wheel defects, which according to Nielsen and Johansson, Attivissimo et al., Johansson and Nielsen [310,311,312] include defects such as wheel flats, out-of-roundness, spalling, and shelling. The advantage of the system described in Wei et al. [37] is that sensors and connecting fibers installed next to the railroad side (**electric sensing device**) are passive to electromagnetic interference from the railroad environment. Therefore, this kind of system does not need any additional power sources, and can be installed at significant distances from the measuring points. The system can be integrated with other applications of railroad fiber Bragg grating sensors, such as the train axle counting presented in Lie et al. and Wei et al. [313,314], for example. The sensors were installed near the foot of the rails, which was considered to be the most sensitive and practical allocation in Wei et al. [314]; therefore, the system might be a bit of a nuisance, especially since the rails must be polished, and pairs of sensors must be installed in the same location to ensure that wheels located on both sides of an axle pass the two measurement points at the same instant. This system consists of real-time cameras and RFID receivers installed for train identification, and results in mapping. Moreover, in Attavissimo et al. [311]—mentioned previously—the authors used a laser diode and a charge-coupled device camera for measurements of wheels and railhead profiles. The conglomeration of such a device allowed us to evaluate the wheel–rail interaction quality. This system recognizes anomalies in wheel–rail contact; however, according to Wei et al. [37], it does not identify the type of wheel or rail defect. Furthermore, systems with devices such as lasers and cameras require precision setup and calibration, and cannot interfere with the extreme track gauge or the vehicle gauge, posing challenges for practical railroad application.

Alemi et al. [315] presented their research on the measurement of wheel diameter as a part of rail vehicles’ condition monitoring, with the use of a commercial monitoring system called the Wheel Impact Load Detector (WILD). This system collected monitoring data in order to detect potential defects in wheels based on the measurements of at least one **strain sensor**, with the ability to monitor more features of the wheel condition using the same system. This system was also used by other researchers, as in Gao et al. and Harrison et al. [146,316], for more complex solutions.

The next keyword, “gyroscope”, was mentioned previously in [50,91,153,154,156,242,263,274,289]; some other publications are briefly described in the next paragraph.

Tsunashima et al. [262] considered a system that can detect cracks, corrugation, and other irregularities in tracks, especially for bullet trains that are the part of the Shinkansen railroad system; this system is called RAIDARSS-3, and can also be used in conventional trains. Within the authors’ system, in-service vehicles are equipped with a microphone, accelerometers, a rate gyroscope, a GPS receiver for positional detection, a computer for analysis, and an analog device transferring signals from each sensor to the mentioned computer—this system was also mentioned previously in Tsunashima et al. [205]. All of the equipment was put inside a compact, portable sensor box installed inside the car body. As the authors of Tsunashima et al. and Kojima et al. [262,317,318] claimed, a set that consists of simple sensors and GPS is sufficient to serve as a probe in order to monitor, detect, and analyze a vehicle during its operation. Furthermore, such a system can detect maintenance problems at an early stage of their occurrence Hayashi et al. [319]. According to Tsunashima et al. [262], the detection of rail corrugation with direct measurement signals (in particular threshold processing) is problematic; therefore, in this system the authors proposed using a microphone for detecting corrugation with spectral peak calculation. Spectral peak calculation was applied to detect rail corrugation based on measurements obtained from cabin noise. Corrugation findings were transformed within the analyzing computer on the basis of multiresolution analysis (MRA), after Daubechies [320], and FFT analysis. The authors did not see any contraindications when using accelerometers to detect track irregularities (in the system, track irregularities were estimated based on incoming signals from the vertical and lateral acceleration of the car body). As Tsunashima et al. [262] mentioned, track irregularities are estimated from the vertical and lateral acceleration of the car body in this system. A gyroscope is used in order to distinguish line irregularities from level irregularities. The results of using this system in the field test (during the research the RAIDARSS-3 system was installed on six N700 series bullet trains) were compared with those obtained by multiple inspection trains collectively known as ”Dr. Yellow”, which run on the Tōkaidō and Sanyō Shinkansen lines, along with another inspection train ”East i”, which runs on the Tōhoku, Jōetsu, Nagano, Yamagata, and Akita Shinkansen lines, and contributes to Shinkansen tracks’ safety. A GPS within the system and a map-matching algorithm are used to locate faults on tracks. As mentioned, the system described in Tsunashima et al. [262] can analyze irregularities, cracks, and corrugation; signals of different frequencies indicate what kind of track failure (of those three mentioned) has been detected.

The next keyword is “heterogeneous sensors”, which occurred in only one publication. Fumeo et al. [321] composed a condition-monitoring system consisting of three modules: a data fusion application, a system integrator, and a visualization system; the latter was referred to as the human–machine interface. The system was developed in order to operate several **heterogeneous sensors** in the railway system. In this time, even more systems with various sensors were developed.

Where the keyword “inertial sensors” is considered, it was mentioned only in Chia et al. [50] so far.

Ward et al. [118] reviewed advanced condition-monitoring solutions for rail vehicle bogies, and investigated vehicle-based sensors for monitoring the running gear, as well as suspension and wheel parameters. The work, in this case, relied on simulations, utilizing model-based condition monitoring and Kalman filters with accelerometer data. The method also required knowledge of the track irregularities as well as accelerometers on all bodies for the best results. The authors of Ward et al. [116] observed that wheel–rail contact causes significant disruption to the operation of the railway network, especially during the autumn. The authors developed a method including a Kalman–Bucy filter in order to obtain an estimation of creep forces in a wheel–rail contact area. The algorithm gathered data with the use of inertial sensors mounted on the vehicle bogie and wheelsets. Creep forces were also considered in Ward et al. [117]; the authors reported dynamic method of the detection of creep forces with inertial sensors installed underneath a rail vehicle. In Ward et al. [19], the authors broadly discussed the use of inertial sensors installed on rolling stock in order to monitor track and vehicle conditions; the authors recalled the appropriate allocation of sensors for track/vehicle condition monitoring. Moreover, the authors discussed standards of data sent in such a system. Aside from potential monitoring opportunities with the suggested system, the authors provided certain operational information for use onboard a train in real time.

Bhardwaj et al. [322] researched a system with **inertial sensors** onboard service trains for the detection and localization of railroad track irregularities. In such a system, noise and reduced signal strength were additionally generated by the geospatial position of the **GPS** receivers and the non-uniform sampling of the inertial sensors. Therefore, interference in the measured signals decreased, leading to higher rates of false positives and false negatives. The authors developed a method to limit the occurrence of such a signal-to-noise ratio decrease.

Mei and Ding [45] took into consideration a method that relied on cross-correlations between heterogeneous measurements from **inertial sensors** mounted on a rail bogie. The authors analyzed vertical primary suspensions of bogies; nevertheless, they claimed that the method might be applied in order to observe potential faults in the lateral direction on primary suspensions, as well as in both the vertical and lateral directions on secondary suspensions. The authors also mentioned that using this method and system enables condition monitoring in other dynamic systems characterized by symmetrical configurations.

In Vanraj Dhami and Pabla [323], the authors claimed that vibration analysis via statistical measures was often used for the diagnosis of gear defects; however, acoustic signals—in the opinion of the authors—were also characterized by huge potential. The authors did not keep away from statistical analyses based on the measurements of vibration sensors. Moreover, they combined vibrational and acoustic signals into a fusion of **vibroacoustic signals** (**sensor data fusion**, **sensor fusion**) that produced more promising results than vibrational or acoustic features individually.

Laser scanning was also among the interests of researchers for railway vehicles (track-accompanying), as well as for the tracks themselves, as is mentioned below.

Kim et al. [245] presented their research on sleepers’ condition monitoring and displacement in particularly critical areas. The system collected data via the use of devices mounted on a vehicle, including **inertial sensors**, **laser sensors** mounted on a bogie, positional sensitivity device sensors, and geophones mounted on the sleeper and connected via a controller area network (CAN) bus. This bus allows the transmitting of data between multiple sensor nodes in the **sensor network**. The authors planned to handle up to 16 sleepers in a critical zone using just one set of the aforementioned devices.

In Reiterer et al. [324], the authors stated that **laser scanning**, as a fast and high-precision method, could become an approved option for the measurement of tracks, overhead lines, clearance profiles, and rolling stock. With the use of such a method, it was possible to obtain measurements from up to several million points per second (high densities of measurements spots), while at the same time it was possible to obtain precise measurement accuracy. The authors described an overview of laser-based sensor systems for measuring railway infrastructure, and highlighted advantages and drawbacks associated with such systems.

The condition of a railway wheel can be analyzed based on wheel profile measurement with the use of automatic systems mounted along a railway track. One of such a system’s advantages is pinpointing bad wheels at an early stage. Nevertheless, measurement data in such a system have to be controlled; therefore, the authors of Asplund et al. [93] developed an assessment method for the validation of individual wheel profile measurements, in order to provide adequate accuracy of the wheel profile measurement data. Wheel measurements were compared by four sensors—one per side of two rails; **laser scanning** was taken into consideration in this research. In Asplund et al. [94], the authors presented a wheel defect detector as a device with which to measure and monitor wheels’ parameters along the track so as to detect faulty wheels, which are one of the reasons for track damage. Wheel defect detectors can catch failures such as flats, large shelling, and severe wheel polygonization.

In the Tōkkaidō Shinkansen line, the acceleration on the car body trains has been measured continuously since the inauguration of the Tōkaidō Shinkansen in 1964 (Arai et al. [325]), in addition to track measurement conducted using track geometry cars. In [326], the author Arai presented a two-dimensional **laser displacement sensor** and measuring device that compensates for the car body acceleration in order to allow the measurement of all parameters of track irregularity in the Shinkansen system’s tracks.

The authors of McCormick et al. [327] examined the interior condition of railway tunnels with the use of imaging and laser scanning-based techniques, which led to rapid data capture. These methods ensured the reduction or elimination of the necessity for track access and visual inspections.

The following keywords are characterized by a small number of publications, yet are nevertheless of qualitative value. These are: “multiple sensors”, “onboard sensors” (this keyword is presented in Section 4.2, since the thematic scope of that section suits it better), “pressure and temperature sensors”, “rechargeable sensor nodes”, “strain gauge sensors”, and “remote sensors” (already presented).

A periodic out-of-round wheel generates excitation frequencies evolving into the resonance of a track and vehicle; polygonal defect detection was used in Alemi et al. [328] in order to study the mentioned aspect. Different polygonal defects were successively recognized with that solution, using **multiple sensors,** and the dominant harmonics were decomposed.

One paper by Somà et al. [329] presented experimental data measured via the application of an onboard unit installed on an intermodal freight train. The device was composed of various kinds of sensors to describe the actual conditions of a monitored wagon—especially a braking system. The sensors used in this monitoring system were as follows: **pressure and temperature sensors** to enable analysis of the brakes’ condition, and an accelerometer for analysis of the wagon dynamics, installed on the wagon’s chassis.

A system of wireless **rechargeable sensor nodes** for the analysis of corrugation problems in urban railways was developed in Lu et al. [143]; this system included electromagnetic-induction-principle-based energy generators and **wireless sensor nodes**, creating a **wireless sensors network**. The system was elaborated for the prediction of the dynamic response of railway tracks branded by rail corrugation.

The next in line is an exceptional keyword: simply “sensor(s)”, their derivatives, and related keywords. As may be suspected, many publications were defined by one of these terms—all of these publications are mentioned in Table 4, while some of them are also given below. Therefore, the publications mentioned up to the end of the current subsection are only a few examples, and do not exhaust the full range of the issue.

The authors of Bruni et al. [26] surveyed the application of **sensors**, electronics, and computer processing technologies to suspensions and running gear; the authors focused on the complementary issues of control and monitoring.

In Ward et al. [119], the authors researched the recursive least squares algorithm; a solution based on linear estimations was suggested because of the observation of the strong discontinuousness of the contact geometry during wheel–rail displacement. The algorithm of wheel–rail contact geometry in a rail vehicle system was discussed based on a system equipped with multiple sensors and high-capacity communication buses, both with high processing capability.

Böhm and Doegen [330] deemed switches to be a critical part of rail infrastructure; they stated that switches were exposed to strong forces during train operation and incidentally extreme weather conditions. Using huge quantities of sensors would be far too expensive; therefore, they developed a system to monitor the condition without the application of **sensors**. The authors noted that external data sources had to be identified and accordingly integrated in order to ensure the necessary information otherwise gained from sensors was obtained, as railway switch and crossing systems are exposed to harsh working conditions that make them susceptible to failures and breakdowns; therefore, these are elements of intensified condition monitoring. Furthermore, the authors of Hamadache et al. [114] considered a model-based fault detection method with a modified residual-based technique applied to electro-mechanical railway switch systems.

In Henao et al. [105], the authors avoided the use of mechanical **sensors** in the diagnosis of mechanical faults in railway traction systems; they demonstrated the noninvasive technique of using a traction motor as a torque sensor through its electromagnetic torque estimation for torsional vibration monitoring (also presented in Kia et al. [107]). Kia et al. [106] presented this solution previously based on research using a device on a reduced scale, in which the experimental setup of the traction part was developed and instrumented based on the extrapolation of real scale parameters. Electromagnetic torque estimation with the use of noninvasive measurement as a resource for mechanical torsional stress monitoring in an induction machine driving system at nonstationary operating conditions was also presented in Kia et al. [108]. Tests and validation of the proposed method were conducted with a squirrel-cage induction machine connected to a one-stage gearbox. Kia et al. [109] claimed that a complex structure and the diagnosis of the electromechanical system based on noninvasive electrical sensors drew special attention from railway traction researchers in recent years. In Kia et al. [109], a real traction bogie was used for mechanical condition monitoring.

An electric point machine is a device for operating railway turnouts, especially from a particular distance. The condition monitoring of such a device was reviewed and discussed in Oyebande and Renfrew [52,53]. An approximate method and a computer simulation to replicate maladjustment with the use of noninvasive **sensors** were proposed by the authors. Zhou et al. [331] proposed the condition monitoring of an electric point machine with approximately 15 separate sensors, either connected directly to the electric point machine, or next to the track surrounding it.

Railway tracks rely on numerous bolted joint connections applied to ensure the safe and reliable operation of both the track and trackside devices. Similarly to other elements of railway systems, manual maintenance of bolted joints is characterized as high cost, disruptive, and unreliable, and can be subject to human error. Therefore, the authors of Tesfa et al. [100] developed a system that automatically measured the clamping force of each individual bolted joint; as a result of their analyses, piezoresistive sensors using the force attenuation method were chosen as the most productive sensors for the purposes of this research. The **strain gauge** and capacitor sensors allowed them to obtain fewer satisfying results (the comparison analyses were performed using design optimization techniques).

In Tsunashima and Mori [38], the authors adapted the interacting multiple-model method to detect faults in running railway vehicles; they used the vehicle model/system for numerical analyses of the given solutions, including the lateral and yaw motions in the case of the rail vehicle’s wheelsets and bogie, and the lateral motion in the case of the vehicle’s body. The authors decided to use **sensors** for measuring the lateral acceleration and yaw rate of a bogie, and the lateral acceleration of a body. 

In Lall et al. [147,148], the authors mentioned that a vehicle’s condition may be monitored against failures with the use of resistance-spectroscopy-based state-space vectors, rate of change, and acceleration—the latter two for the state variable. The authors mentioned that actual prognostic health management application areas include fatigue crack damage in mechanical structures—such as those in rail structures—mostly with various types of **sensors**.

In Ngigi et al. [6], the authors appraised the up-to-date condition-monitoring techniques applied for railway vehicle dynamics by analyzing various methods’ advantages and disadvantages. The authors identified wheelset condition monitoring, and track-based **sensors** for the effective condition monitoring of vehicles. They considered the challenge of finding proper measurement technologies, along with relevant and correct parameters that can be measured to meet the expected aims of such systems.

The experiments presented in Czechyra and Firlik [132] were carried out via numerical simulations for different tram types, conducted with consideration of selected faults, under various run parameters—such as speed, vehicle load, track type, etc. The system developed by the authors took into consideration analyses of metrics such as suspension condition, wheel surface wear, ride comfort and stability, and safety against derailment. The system was equipped with a communication module, a positioning device, and a set of **sensors**. Each measurement of sensors located on the vehicle was compared to the nominal values and historical measures from the same sensor (comparison of accelerations and position signals measured during runs over the same track section).

The authors of Thakkar et al. [332] presented a prototype system for predetection of wheel defects using rail-mounted sensors; the authors concluded that their system might be used for the analysis of wheel defects by using either track-mounted or wheel-mounted sensors, after selected calibrations.

In Matsumoto et al. [203], the authors developed a then-new method of measuring wheel–rail contact forces, which was based on non-contact gap **sensors** placed on parts of the bogie in which no rotating parts are situated. The method and the system were verified on the subway/metro line. The authors obtained some important characteristics of wheel–rail contact mechanics (i.e., derailment coefficients, fluctuations of the coefficients being similar on straight and curved tracks) during the system exploitation, with mutual influence.

Chudzikiewicz and Stelmach [333] described a complex solution for monitoring the technical condition of a vehicle and track at the same time; the system described by the authors wasintended to consist of a network of sensors mounted on a selected part of a rail vehicle. The data acquisition unit and a data server—both enriched by software dedicated to the analysis and management of diagnostic data—were also included within the system. The data—i.e., acceleration signals obtained during runs over critical parts of the track section (e.g., crossings)—were intended to be compared with one another. 

Limitation in sensor quantities is important, for example with respect to costs or the sending of signals. Kirkwood et al. [334] discussed such a topic, with reference to railway transport as well; in the opinion of the authors, adding **sensors** is not a difficult task; however, associated costs are connected to those sensors—such as the cost of each sensor, the cost of utilities required to power them, the cost of data gathering and processing, and the cost of storage of that data. These costs must be taken into consideration—especially currently, when wireless sensor networks are of huge interest for various purposes.

In Groos et al. [335], the authors presented a quasi-continuous condition-monitoring system that was developed to analyze short-wavelength (from a few centimeters to a few meters long) defects of railway tracks, such as rail corrugation. The measurements were obtained in this research using a vehicle-embedded sensor as a triaxial axle box accelerometer. The acceleration sensor data were combined with a digital map of linear railway infrastructure and other crucial operational data, which were implemented within a land-side data management system. Track-selective georeferencing by multi**sensor fusion**, the axle box acceleration data applied for pattern recognition, and further intelligent data analysis were used for the temporal condition of track analyses.

Ning et al. [336] took into consideration the problem of small-amplitude hunting detection before the occurrence of lateral instability observed in the case of high-speed trains. Data obtained with only one sensor were considered by authors to be insufficient; therefore, in order to improve the accuracy and robustness of the monitoring system for the detection of small-amplitude hunting, the authors developed a multi**sensor fusion** framework using the Dempster–Shafer theory. This multisensor fusion framework consists of several steps, described in the paper in detail. The authors suggest that the mentioned framework can also be used as a gearbox monitoring system, since nonstationary signals are acquired.

In Xu et al. [264], the authors proposed a method of wheel condition monitoring in the context of their wear; the method was developed in order to provide analyses for high-speed trains’ wheel wear using onboard **vibration sensors**. According to the authors, techniques and statistical methods for signal processing were applied in order to obtain multilocation vibration data and estimate the wheel wear.

Roth et al. [337] suggested a system for track condition monitoring developed via the **sensor fusion** (**sensor data fusion**) of a global navigation satellite system (GNSS) and inertial measurement unit (IMU) measurements, together with a railway network map. This system is characterized by an offline positioning problem; therefore, the authors proposed positioning estimation by a two-stage approach based on GNSS data and a railway map. These approach stages are determination path hypotheses from the GNSS positions at the first stage, whereas the second stage is connected to smoothing extension to the Kalman filter, i.e., nonlinear Rauch–Tung–Striebel-smoothed position and speed estimation. The measurements of railway track coordinates with the use of the GNSS technique were also applied in Wilk et al. [338]; the authors assessed the quality of obtained measurements, and investigated the potential for their further processing. Their aim was to develop the usage of geometrically constrained parameters of the base vector, along with specialized digital filtering applied within numerical analyses connected to the research, in order to precisely determine the track axis.

Wolf et al. [17] suggested a concept of a tram condition-monitoring system in which a meshed and **wireless sensor network** can procure vibration measurement data. The concept was based on the assumption that sensor nodes were event-triggered, in an energy-efficient manner. The system would evaluate decentralized data collected by meshed and autonomous **vibration sensor** nodes.

An unmanned high-speed vehicle was studied in Gong [339]; the author presented an anomaly detection method with the use of an acceleration **sensor** on an axle box. This method’s author proposed the use of a quasi-double-layer chloroprene rubber filter, instead of an algorithmic solution, to isolate the high-frequency component of the shock acceleration signal. Apart from the vibration acceleration of an axle box of a vehicle, the device accompanying this method consists of a data acquisition card and a train speed sensor. The author described changes in the statistics of particular parameters connected to condition monitoring in detail. The author concluded, for example, that differences in vibration suppress characteristics of an axle box damper, and that the use of response ratio to detect the abnormality of the axle box damper can determine the abnormal condition of the axle box’s acceleration (detection of fault) of the unmanned vehicle.

In Barman and Harazika [340], the authors attempted to develop a **sensor**-based system to achieve train condition monitoring; this system was a **vibration sensor**, together with the insignificant effect of temperature on its measurements. In order to store vibration data, an embedded system using the Arduino Uno board was developed. The authors observed that the time domain analysis provided information on the slip and derailment tendencies of a train, while frequency domain analysis indicated the condition of various train components.

### 4.2. Analysis of Various Types of Sensors Used in Different Solutions Connected to Wireless and Online Communication 

As mentioned in the final part of Section 3, an analysis of selected papers related to wireless and online communication used in different solutions connected to condition monitoring in railway transport is presented next. The list specific keywords for these papers is given in Table 4, starting with “Bluetooth communication”, through “GPS”, “map-matching algorithm” (already presented in the previous subsection), “smartphones”, and plenty of similar keywords connected to wireless communications and wireless networks—such as “wireless sensor network”, “wireless sensor network (WSN)”, “wireless sensor networks (WSNs)”, “wireless sensor node”—as well as “artificial intelligence” and “big data”.

In Daadbin et al. [341], the authors presented a condition-monitoring system in which components allow the monitoring of both axle vibration and pantograph load. The monitoring system consisted mainly of strain gauge instrumentation, telemetry systems, and data loggers. The measured data—such as torque and bending moments on a gearbox shaft, and load or actual acceleration on a pantograph—were continuously monitored and diagnosed during the trains’ routine operations. Rapid and accurate identification of the location and exact time and date of an alarming event were provided by **Bluetooth radio communication** with trains and their onboard GPS systems. This system was also described in a previous paper by Daadbin and Rosinski [342] (especially in the context of a numerical model for the traction system), and it was mentioned that it used two subsystems—namely, a digital processing module, and a receiving signal and transmitter unit, both installed inside the carriage.

The device designed and presented in Mizuno et al. [343] consisted of a **GPS** receiver, an accelerometer, and an analog–digital converter. A sensor was settled on a vehicle floor, as opposed to axles or bogies as in the case of numerous other studies. The authors recognized such a location as being effective for capturing vertical track displacements, and the measurement of acceleration response on a vehicle’s floor as a promising index to capture railway track disorders in the vertical direction. The authors’ aim was to trace a route and, at the same time, collect the acceleration response of a passenger vehicle. 

In Lo Schiavo [344], the authors presented a **wireless**, energetically autonomous **sensor network** for the onboard monitoring of railway freight wagons. The energy harvester used in this solution ensured independent networks of **sensor nodes** for each wagon. Reduction in energy consumption without worsening quality of service was assured by the use of a bi-periodic communication scheme for the local wireless transmission: a **GPS** receiver, and a GPRS transceiver; the author called this “optimized management of the sleep modes”.

In Aimar et al. [345], the authors presented a condition-monitoring system for an intermodal freight wagon. The system was powered by a battery, as most freight cars are not powered by traction sources of electrification. The authors mentioned that the main aim at the beginning of their research was to enrich a database for the development of a diagnostic algorithm for an embedded remote-monitoring system based on monitored parameters, which were: **GPS** position, pressures of the pneumatic circuit of a braking system, the temperature of a brake hanger and blocks, and onboard acceleration recorded during train operation. 

Zoccali et al. [346] assessed ride comfort inside rail vehicles according to ISO 2631. For this purpose, the authors computed vertical-frequency-weighted acceleration for subsections of fixed lengths, and commensurately mapped the obtained measurements on geographic information systems (data were gathered on railway lines characterized by automatic train operation). This enabled the authors to assess whether discomfort occurred due to localized irregularities or worn rail switches. The system the authors used consisted of an inertial measurement unit (IMU) integrated together with a **GPS** receiver.

In order to enable the use of smart technologies with GPS in the condition monitoring of freight railcars, an effective power source is required. In Pan et al. [347], the authors developed a freight railcar electromagnetic energy harvester at the secondary suspensions, with a mechanical motion rectifier mechanism. The harvester was designed, modeled, and tested in both laboratory and field conditions. The paper presents the design, modeling, in-lab tests, and onboard field tests of the energy harvester. According to the authors, this energy-harvesting device can acquire the vibrational energy that is usually dissipated or wasted.

Davies [348] mentioned that the rail industry was unwilling to respond to the introduction of smartphones and tablet computers, which has changed recently. In Aung et al. [349], the authors considered onboard sensor measurement and satellite image analysis applied for the early detection of potential deterioration of track condition. The authors used an accelerometer in a **smartphone** (**onboard sensor**) in order to measure acceleration in the vertical and lateral directions. The smartphone was placed against the car body; the authors assumed that a smartphone vibrates when passing irregular rail track sections.

In Seraj et al. [350], the authors worked on a system called RoVi, which was a **smartphone**-based framework for the continuous monitoring of numerous condition indicators for infrastructure conditions and assets. The system used data acquired via smartphone **GPS** and the **inertial sensors** of devices carried by train passengers to provide actual space-time information on monitored infrastructure (the crowdsensing assumptions were applied). The data were processed by an optimized processing algorithm, which led to computing indicators connected to railroad track geometry features such as a cant, a twist, a curvature, and an alignment for different segment lengths, as well as a path roughness index.

Bridgelall and Tolliver [351] noticed, as with many other researchers, the necessity of lower cost and more scalable solutions for sensors used in onboard rail vehicle monitoring approaches. These solutions are generally associated with a great deal of uncertainty, especially considering that various aspects of anomalies that occur—such as potholes, frost heaves, swelling, and cracking—can rapidly progress with an increase in dynamic vehicle loading and changes in weather cycles [351]. Therefore, researchers used **smartphones** as devices that might be useful in order to evaluate a variety of methods and applications used in rail vehicles. The authors underlined demanding aspects connected to geospatial positioning system receivers. The experiments conducted in this research found that the longitudinal **GPS** error spread ranged from 21 m to 32 m (or sometimes even exceeded this value), while the error in lateral direction spread ranged from 9 m to 15 m. Moreover, according to the authors, the error connected to geodesic distance increased linearly with traversal speed, from 16 m at speeds of 18.8 m/s to 27 m at highway speeds of 31.3 m/s. The authors opened the door for future deliberations on using such systems’ positioning.

Two types of special equipment vehicles are mentioned in the literature: one of them is the track recording vehicle (TRV), which is used for track inspections in order to maintain the safety of the tracks, while the other is the hauled track recording coach (TRC), which collects track geometry data, Chudzikiewicz et al. and Weston et al. [176,238]. In Balouchi et al. [352], the authors claimed that in the UK, TRVs were used; however, they developed a low-cost Tracksure track-monitoring system. This system is dedicated to the detection of track voids and other track defects and irregularities (Balouchi et al. [352] claimed that the system can also determine corrugation and wheel flats); it makes use of the existing onboard GSM-R cab radio operated in every single UK train, through the fitting of a sensor card that detects track condition over three axes of train vibrations. Identification of voids is realized by acceleration signals measured with the use of sensor cards installed within the vehicle cabs of multiple trains to assess each track section. In this system, the machine reads data packets computing the acceleration signal’s root mean square (RMS). Additionally, the system sets the RMS thresholding dynamically, based on machine learning from the multitrain analysis; the data are obtained using a **GPS** system.

In Bosomworth et al. [112], the authors considered the situations in which a particular parameter cannot be measured directly on a spot connected to it—such as rolling contact, for example. At the same time, the authors took into consideration the fact that a device operates in extreme environmental conditions. They stated that simulation modeling plays a significant role in such circumstances; therefore, the authors integrated a multibody simulator into an onboard passenger vehicle device with local sensors, such as GPS, in order to send an input signal to a simulator that computed indicators of wheel–rail contact and derailment risk.

In Bogue [353], the author briefly mentioned the application of a wireless-based stress-monitoring system that was dedicated to the monitoring of railway tracks and rolling stock; however, other large structures were also mentioned in this paper.

Hodge et al. [32] surveyed **wireless sensor network** (**WSN**) technologies for railway transport condition monitoring. The authors analyzed systems, structures, vehicles, and machinery in the context of **wireless communication**—they were especially interested in solutions for the monitoring of infrastructure (bridges, rail tracks, track beds, and track equipment) along with vehicle health monitoring (chassis, bogies, wheels, and railcars).

In Krichen et al. [354], the authors researched optical wireless sensor network architecture based on visible light communication and free-space optics technologies. In order to ensure rail transport traffic safety, light-based sensor nodes were proposed. Such sensor nodes allowed the authors to measure the amplitude and the frequency of the rail vibration during a train’s operation. Optical sensors located at the rail foot used visible light communication to transmit appropriate data. Free-space optical communication was used to enable contact between gateways, and enabled communication between optical sensors.

In Maddison and Smith [355], the authors presented a solution that supports wireless sensor networks—especially for rail tunnels, track beds, and track monitoring—during its construction and future exploitation. This solution was described in the specific context of tunnel deformation monitoring during engineering works. Moreover, the system ensured the ability to measure fluctuations in track cant and twist, as well as the longitudinal rate of change. These measurements were collected with extremely high precision and stability, which the researchers attributed to sensor nodes. The wireless solution is believed to be superior to those based on optical sensors. The authors mentioned that the measurements and data obtained were highly stable and repeatable, without any need for frequent maintenance or configuration of reflectors, without susceptibility to noise, and—since wireless sensor networks were used—the system avoids the need for wiring. This battery-powered system (battery life extended to over 10 years) ensured high precision, Maddison and Smith [356].

The authors of [23] reviewed various types of wired sensors and sensors in **wireless sensor networks** (**WSN**) that were used for the condition monitoring of railway tracks. The authors described the application of particular sensors in order to monitor the performance of different track components, especially during unpredicted occurrences. 

In Nsabagwa et al. [357], the authors presented a condition-monitoring and reporting structure, which relies on a wireless sensor network with sensor data to establish automatic weather station health. This kind of solution might also be used as part of a transport system; therefore, it is also mentioned in this paper. Based on the identified problems in a database, SMS or email was sent to the concerned person.

In Hussels et al. [358], the authors researched a condition-monitoring system that reveals trace threats in pipes with **fiber optics** (**optical fibers**); however, the authors mentioned that the great potential for distributed acoustic and vibrational sensing was also rapidly evolving in train and track monitoring, e.g., in Lienhart et al. [359]. The subject of **optoelectronic and photonic sensors** used for the condition monitoring of pipelines was considered in Lopez et al. [360]. In Lienhart et al. [359], the authors reported certain approaches to continuously monitoring railway tracks and vehicles by using distributed **fiber-optic** sensing techniques. The monitoring system consisted of fiber-optic strain-sensing cables that were located along railway tracks, and strain changes due to rail deformations were depicted by distributed Brillouin measurements. Such measurements allow the detection of possible damage to the railway infrastructure due to natural causes such as mudflow, avalanches, floods, and landslides, and according to the authors can also head off secondary damage by enabling fast and appropriate counteractions. The authors used optical communication cables that had already been allocated along rail infrastructure to detect flat spots in railway wheels. In summary, the authors monitored both incidents and deformations.

**Wireless sensor networks** do not require regular replacement of batteries. In Narampanawe et al. [361], the authors found an energy harvester to collect energy from current-carrying cables available within railway systems and building facilities. The authors explored an ultra-thin inductively coupled transformer that may be used for energy collection purposes to power up sensor nodes. The authors described that this solution may be manufactured on a flexible printed circuit board in the form of an autonomous **sensor node**.

These systems in Gao et al. [142,144,145] are called self-powered **wireless sensor networks** for **wireless communication**. The condition-monitoring system based on energy harvesting was described in the abovementioned paper Gao et al. [142]. A track-side, vibration-based, electromagnetic-energy-harvesting prototype was previously analyzed in Gao et al. [144]; however, this system was designed for track analysis only. The system described in Gao et al. [144] provided power to rail-side monitoring devices connected to sensors that ensured the collection of wheel–rail vibration energy during the train rides. This prototype was equipped with **wireless sensor nodes** coupled with an accelerometer and a temperature/humidity sensor. In Gao et al. [145], the authors extend an energy-harvesting system with a self-powered ZigBee wireless sensor node as a coordinator of an accelerometer, a temperature sensor, a humidity sensor, and an infrared detector.

Energy harvesting was also of interest in Espe and Mathisen [362]; the authors evaluated the implementation ability of energy connected to the magnetic fields, in order to increase the lifetime of distributed condition-monitoring systems. This energy was intended to be gained near electrified railway tracks. According to the authors, it may be used for low-power operation and low-duty-cycle **wireless communication** in the case of condition-monitoring systems.

In Cii et al. [363], the authors presented a system for the condition monitoring and structural diagnosis of freight railway vehicles. The authors presented the design, and the laboratory test results of **wireless sensor nodes** (**WSNs**) equipped with three energy-independent and autonomous onboard production systems based on solar, wind, and mechanical vibration power available during trains’ operation. The three sensors, using various energy sources, were compared according to their fatigue behavior and performances during laboratory tests.

### 4.3. Current Aspects of Industry 4.0 Applied in Condition Monitoring in Railway Transport

As noted in the final part of Section 3, an analysis of selected papers connected to selected aspects of Industry 4.0 applied in condition monitoring in railway transport is presented below. The list of these review papers’ specific keywords given in Table 4 starts with “artificial intelligence” and “big data”, proceeding to “Industry 4.0”, and through “intelligent condition monitoring”, “intelligent systems”, “Internet of Things”—and also “Internet of Things (IoT)”—and finishes with “robotics”.

Numerous abovementioned publications presented extensive condition monitoring, signal gathering, and signal collection; nevertheless, the application of deep learning and artificial intelligence approaches applied to the detection, diagnosis, and prediction of faults was limited.

Yella et al. [290] summarized the research and outcomes of a large number of studies connected to **artificial intelligence** techniques applied to the automatic interpretation of data from nondestructive testing carried out on rail inspection problems; certainly, the authors’ review was pertinent at that time.

A specific subject of research was taken into consideration in Wu et al. [364]; the authors analyzed the dynamic changes in vibration radiation noise in a bullet train’s passenger compartment luggage rack. According to the authors, a three-dimensional spectrum is sensitive to changes in train operation status, operation speed, and track condition; therefore, their method can be extended to noise measurement and analysis of the passenger compartment. The authors mentioned that this method can be used for the measurement and analysis of both passenger compartment noise and exterior noise. Moreover, the authors suggested that this method can be applied for train operation state monitoring and comfortable evaluation of passenger compartment noise.

The authors of Saeed et al. [365] discussed aspects of condition monitoring and fault diagnosis in a Francis turbine. The authors integrated numerical modeling with several different artificial neural network techniques (**artificial intelligence**). This research was not directly connected to rail transport condition monitoring; nevertheless, it was indexed in the database under the matter of interest, and it can be applied, especially considering that the authors mentioned that the multiple adaptive neuro-fuzzy inference systems technique was applied for monitoring and diagnosis in different practice areas, as well as for railway track circuits in Chen et al. [366].

In Marwala [367], the author described **artificial intelligence** methods; among these methods, the author included neural networks, particle swarm optimization, genetic algorithms, and simulated annealing. 

The authors of Su et al. [127] developed linear dynamic, hybrid-model predictive control, which was used to describe the evolution of railway track segment’s condition. The authors of this model provided a detailed procedure to illustrate how to transform the model-based optimization problem into a standard mixed-integer quadratic programming problem. For future work, the authors planned to enrich this model with parameters obtained from track measurements. According to the database, this research was connected to **artificial intelligence**.

Another study connected to the use of **artificial intelligence** was connected to thermal camera image processing. In Yaman and Karakose [175], the authors analyzed the detection of rail surface defects based on complex fuzzy automata. The authors applied Otsu’s method for images taken from thermal cameras. The obtained results were used as the input values of the complex fuzzy automata system. According to the authors, the proposed method was not affected by environmental conditions when using a thermal camera. Their research’s success ratio in the detection of rail surface defects was almost 90%.

In Sun et al. [368], the authors presented an approach for the detection of rail joint failure on a railway track based on acceleration measurements by training convolutional neural networks (**artificial intelligence**). The system detected joints or failures, and was able to detect failures on both rails using one model. The developed system worked with raw data input in order to obtain a reduction of heavy data preprocessing. The experimental results showed that both convolutional neural networks—namely, ResNet and FCN—obtained satisfactory performance.

In Mosleh et al. [369], the authors presented an approach that allows the identification of wheel defects and wheel flats with the use of a wayside monitoring system. To do so, a methodology of envelope spectrum analysis was applied (artificial intelligence included, according to the database). The authors presented tests, and discussed and analyzed the sensitivity of the proposed approach to track irregularities, randomization of both the positions and size of a wheel flat, and perturbation resulting from different noise intensities.

Fink et al. [5] provided a thorough assessment of actual developments, drivers, challenges, solutions, and planned studies in the field of deep learning and **artificial intelligence** approaches applied to prognostics and health management (PHM) applications, taking rail transport into consideration as well. The authors, for example, mentioned publications of Gibert et al. [370] that described the use of image data collected in order to build a multi-task deep convolutional neural network for railway infrastructure monitoring. The elaborated convolutional neural network (CNN) was able to recognize elements of railway infrastructure. Moreover, the application of the CNN allowed the detection of damage, such as cracked concrete ties and missing or broken rail fasteners.

Niebling et al. [371], staying in the mainstream of low-cost sensor applications—e.g., Knight-Percival et al. [372]—on in-service trains, suggested deep learning (**artificial intelligence**) in order to analyze large volumes of sensors’ measurement data. The authors combined common signal processing methods and deep convolutional autoencoders and clustering algorithms. Such a combination of methods and techniques was applied to find various rail track anomalies and their patterns; further improvements and developments were planned.

In Núñez et al. [126], the authors observed that countless railway track condition-monitoring data (terabytes of data from various sensors) were being collected, yet not fully used, because of insufficient suitable techniques to extract the relevant events and crucial information. The authors discussed conditions of the five Vs of **big data** in railway condition-monitoring systems, namely: volume, velocity, variety, veracity, and value. The authors described an axle box acceleration measurement system called the ABA system, which incorporates failure and maintenance information in order to be adaptive and self-learning.

In Lin et al. [257,258], the authors suggested applying **big data** analyses in the condition monitoring of railway systems, since these monitoring systems enable big data to be accumulated during commercial operations. In this research, the authors use a naive Bayes classifier as a method of big data analytics, which helped to identify individualities of railway vehicles.

In Lin et al. [256], the authors described an on-track condition-monitoring system to measure the wheel load and force of railway vehicles generated in the lateral direction, in order to obtain both vehicle and track inspection information after **big data** processing.

Lin and Suda [255] developed a method implemented in the on-track condition-monitoring system that assists in the detection of a vehicle’s abnormality by adding both vehicles and track inspection information within **big data** processing. This method was implemented in the monitoring system for the wheel load of railway vehicles, and for force generated in the lateral direction. The authors’ results aid in the determination of whether or not vehicle and track inspection were executed. 

In Grossoni et al. [373], the authors analyzed track stiffness as a key physical parameter for track quality assessment and long-term track deterioration. The authors verified the role of track stiffness and aspects of its spatial variability through a set of experiments based on digital information and **big data**. The authors’ analyses were a kind of sensitivity assessment, with other vehicle and track physical parameters being under quantitative alteration (the following parameters were varied: vehicle unsprung mass, speed, and track vertical irregularities; data connected to stiffness and vertical irregularity were analyzed using a sample procedure based on an ARIMA stochastic process). Track geometry deterioration rates were estimated based on a vehicle–track interaction model, which was coupled with series of log-linear regression models applied to analyze the impact of variable parameters on track deterioration. According to the authors, spatial variability of track stiffness was characterized as having a significant impact on track deterioration rates.

Pistone et al. [374] discussed two varied series of results of a continuous monitoring system of the operating track: firstly, for massive train wheels, and secondly by impulse tests performed in nine selected stations of the underground in Vienna. The reason for the test was the substitution of conventional resilient wheels for massive wheels in order to increase the performance of vehicles, reduce operational costs, and increase safety. Selected trains circulating in the underground network were equipped with massive wheels, and modules of the system were fitted with **inertial sensors**. Nine measuring systems were installed within the network in the form of monitoring devices applied to continuously record data. Radio frequency identification tags (**Internet of Things** employed) were installed for train identification, and the information on vehicles’ condition—and especially data on the impact of massive wheels—were sent to headquarters. Three years of this testing allowed analysis of the **big data** used for real-time diagnostics of infrastructure.

In An et al. [9], the authors researched a recurrent neural network with a long short-term memory in order to diagnose faults in motor drive systems characterized by **big data** measurements. The authors wanted to exclude the limitations of existing intelligent methods—namely, the fact that the training data and testing data are subject to the same working conditions in such methods. They developed a novel three-layer model inspired by a recurrent neural network coupled with transfer learning, and verified the proposed method using a rolling-element bearing dataset. The researchers’ results were an improvement in comparison to artificial neural networks and sparse filtering. The authors’ methods ensured rapid judgment of the condition of the analyzed elements.

In Xu et al. [375], the authors defined abnormal segments in condition monitoring that not only reduce the quality of data for condition monitoring and big data analysis, but also bring a heavy computation load. The authors mentioned that these segments are missing segments and drift segments, which occur unavoidably in big data analyses when sensors undergo failures, network segment transmission is delayed, or there is accidental loss of some collected data, etc. Therefore, the authors elaborated an abnormal segment detection method in order to improve the quality of **big data**. The solution was verified with actual wind turbine data; however, this method might be used for the condition monitoring of rail transport machinery.

In Xu et al. [376], the authors developed an overview of predictive maintenance systems, with a particular emphasis on existing models, methods, and algorithms ensuring data-driven fault diagnostics and prognostics. The authors elaborated such an overview because of the massive growth in the research of condition-monitoring data of industrial equipment (industrial **big data**). When rail transport was considered, the authors mentioned de Bruin et al. [377] as a study on a long short-term memory recurrent neural network that was applied to learn from dependencies on raw railway track circuit data and their diagnosed faults.

In Saki et al. [378], the authors suggested a discontinuance of onboard data computing with the use of signal processing coupled with enriched machine-learning-based approaches. The authors’ reason for such a decision was to limit the transmission bandwidth on the public mobile networks. The researchers developed a device consisting of two main parts: the first part was a data classification model that classified **Internet of Things** (**IoT**) data into maintenance-critical data and maintenance-non-critical data, while the second part was a data transmission unit ensuring communication methods to transmit **big data** to railway control centers. For the transmission of maintenance-non-critical data, the authors proposed a specific method that employs train stations as points at which data are transferred and delivered to mentioned centers. As this solution concerned passengers’ stations, it could therefore be applied only in the case of passenger trains.

Parhi et al. [379] developed an inference system of multiple adaptive neuro-fuzzy characteristics, and a methodology related to the detection of cracks in structures. According to the authors, this methodology can be applied for the condition monitoring of dynamic structures, such as railway structures; nevertheless, the authors did not conduct research on rail structures directly. The authors mentioned that the authors of Chen et al. [366] studied fault detection and diagnosis for railway track circuits with the use of neuro-fuzzy systems.

In Molodova et al. [129], the authors presented the results of rail joint condition monitoring based on axle box acceleration measurements. The authors considered measurements obtained from trailing and leading wheels, in the case of both the right and left rails. The obtained characteristics aim to obtain knowledge of the quality and the state of the insulated joint in order to develop intelligent preventive maintenance strategies (**intelligent systems**). The authors validated their monitoring method by in-person visual track inspections and hammer tests of the insulated joints.

A vehicle-borne battery is one of the pieces of equipment used in magnetic levitation trains, whose condition highly affects the efficiency of the train’s operation. In Zhang et al. [267], the authors developed a **sensor network** designed to monitor batteries’ condition in each car—especially the batteries’ temperature and remaining capacity. This solution is based on the **Internet of Things** concept, thanks to which a train operation control system collects measurements of all cars in a maglev train by using its communication network through the radio communication system. Furthermore, in Gao et al. [145], the authors benefitted from the development of the Internet of Things in the area of intelligent railway transport.

In Sramota et al. [380], the authors referred to an autonomous, near-real-time system for the condition monitoring of railway tracks, points, and crossings; the system works during the operation of a nearby train. Sensors or sensor nodes created **wireless sensor networks**. As the authors mentioned, the aggregation of data was realized with a low-power, wide-area, Internet of Things network structure. Such a network provided post-processing big data.

Karaduman et al. [65] presented a method based on images taken from the cameras monitoring the railways. These images were transmitted to a central computer using the concept of the Internet of things (IoT) as a technology continuously applied for condition-monitoring systems (including artificial intelligence). The authors applied image processing techniques to these images, and afterwards meaningful data were extracted in the central computer. This system was developed for contactless fault diagnosis—especially for pantographs and catenary. It should be mentioned here that, although this review paper is mostly concerned with conventional vehicles and tracks in railway transport, the other solutions and their condition monitoring are getting to a more rapid stage. One such solution is the concept of magnetic levitation (popularly called maglev) coupled with solutions standardized for the concept of Industry 4.0, making the pair an example of intelligent transportation. In Sun et al. [381], the authors developed a method for the control of suspension for medium–low-speed maglev trains. The authors incorporated the **Internet of Things** (**IoT**) technology and an adaptive fuzzy controller (developed by the authors). Firstly, the authors established a mathematical model for the analyzed suspension system of the mentioned trains, and then they composed the IoT and the circuit design. Based on historical data from previous rides of such maglev trains, the authors extracted suspension airgap control laws, and as a result they developed adaptive fuzzy rules of maglev trains’ suspension systems. All of these operations resulted in the verification of the method’s effectiveness by the testing of full-scale maglev trains. The authors stated that for future results they intend to focus on the tuning rules of the proposed controller in order to extend it to other systems.

Chen et al. [252] supported maintenance activities with **intelligent condition-monitoring** systems that ensure availability, reliability, and safety in railway operations. The authors’ proposed method uses a combined technique of quantitative and qualitative characters, known as a coactive neuro-fuzzy inference system, for the detection and identification of the 10 most common failures of railway track circuits. They applied a single audio frequency jointless track circuit for the verification of the simulation model, and for collecting fault data. 

Sandidzadeh and Dehghani [382] employed a neuro-fuzzy network (coupled fuzzy systems and neural networks) for fault detection and identification in a typical audio frequency track circuit, which is a hybrid of knowledge-based and data-based systems. This kind of research is related to **intelligent condition monitoring**. The authors’ solution was generated for interaction with low-precision data, and it is characterized by the learning ability of a neural network. The method was verified by analyses of modeled and simulated regular track circuits. Data with and without faults were used for training the algorithm of the neuro-fuzzy network. 

In Anyakwo et al. [83], the authors developed a two-dimensional wheel–rail contact model, with the exclusion of some variables of the contact model’s geometry in order to reduce computational time. A lookup table was applied to ordinary differential equations representing the dynamic bogie behavior, and as a result computation time was shorter. This method can be applied for fast and real-time simulations of hybrid **intelligent systems** (hybrid, according to the authors, consists of mathematical modeling and computer simulation techniques). A novel 2D wheel–rail contact model was applied in this method.

In Etchell et al. [383], the authors developed a remote system for condition monitoring, prediction, and prevention in case of failures of underground jointless track circuits. Such a solution allowed the analysis of a large-scale, geographically diverse, **intelligent system** that can conduct real-time condition analysis of voltage and frequency for circuits.

Márquez et al. [214] coupled **intelligent condition monitoring** with reliability-centered maintenance, which allows the collection of both digital and analog signals obtained from transducers connected to either point-to-point or digital bus communication links, depending on signal form. Their research conducted failure analysis, data acquisition, and incipient fault detection on a hydraulic railway level crossing barrier system. For each barrier, not only the hydraulic characteristics, but also the motor’s current and voltage, hydraulic pressure, and barrier’s/rotary’s position were acquired. Data without errors were obtained with the use of a single-cable fieldbus that enables all sensors’ connection through appropriate communication nodes to a bus characterized by high speed. The researchers used ARIMA (autoregressive integrated moving average) models for the detection of faults. The models were tested accordingly with Jarque–Bera and Ljung–Box statistical tests.

A Bayesian network related to the intelligent condition monitoring of railway catenary systems was developed in Wang et al. [122]. This network’s parameters were learning based on historical data. The authors suggested the following types of measurements for considering the mentioned monitoring systems: wire stagger, contact wire height, pantograph head displacement, pantograph head vertical acceleration, and pantograph–catenary contact force. The authors were curious about both the physical meanings of the mentioned parameters and the correlations between them. The research resulted in catenary condition level identification. 

As Joshi et al. [46] mentioned, an intelligent condition-monitoring system requires a proper data storage system for the detection of incipient failures reported during the condition assessment of distributed generation systems. Such systems are also used in rail transport. The authors currently surveyed used applications and various soft computing methods for condition monitoring in the context of **intelligent systems** used for diagnoses of distributed generation systems.

Among the less typical—yet still important—research problems from the cities’ point of view, the cleaning of light rail transport areas can be considered. In Dong et al. [194], the authors developed a road–rail precision cleaning vehicle designed to clean the groove rails of modern tramways. The authors developed an overall mechanical structure for such a vehicle, as well as an electrical control system with a high degree of automation and, moreover, they proposed a condition-monitoring system and human–machine interface system. Combining both a programmable logic controller and a touchscreen industrial personal computer allowed them to realize an automatic **intelligent system** for cleaning based on image recognition that coupled with automation, improved the efficiency of cleaning the rail and reduced—for example—water consumption, which is used for cleaning. The authors planned to continue using image recognition technology in order to improve the classification of garbage by deep learning with image recognition. 

In Sun et al. [384], the authors developed an **intelligent condition-monitoring** method for railway point machines (equipment that enables the switching of routes for trains) based on sound/noise analysis. The authors used the following methods and techniques: particle swarm optimization, support vector machines, k-nearest neighbors, random forests, and naive Bayes classifiers; support vector machines were stated to be the best of this group for identification accuracy and computing cost.

As Karakose and Yaman [174] mentioned after Aazam et al. [385], **Industry 4.0** technology provided the development of the Internet of Things (IoT), big data analytics, smart machines, cloud-based manufacturing (CBM), and cyber-physical systems (CPSs). The authors developed a complex thermography method coupled with fuzzy methods for the railway and pantograph catenary system, which resulted in an approach for predictive maintenance on electric railways that relies on a complex fuzzy-system-based thermography. A coupled approach using thermal image and signal processing methods was applied by the authors. The reason for the proposal of the fuzzy approach was that it was suitable as a maintenance estimator when seasonal weather conditions, external environmental conditions, daylight, and especially intermittent effects such as train speed were taken into consideration. Moreover, the authors mentioned that thermal changes impact both rail surface and pantograph catenary; therefore, they suggested the coupling of fuzzy and thermal image processing methods. Moreover, in Knight-Percival et al. [372], the authors observed that the interdisciplinary field of systems engineering—in particular transportation systems—is extended and supported by **Industry 4.0**. Industry 4.0, according to the authors, focuses on the design and management of complex systems during their life cycles, with consideration of four design principles—namely, interoperability (communication and connection between systems, devices, and sensors), information transparency (integration of sensor data with a digital twin of a physical system), technical assistance (visualization of complex data and reduction of workload), and decentralized decisions (autonomy of complex systems that can adapt to the specific environment). In Knight-Percival et al. [372], as a consequence of the implementation of such principles, the authors developed a custom-built, low-cost magnetic field detection system for track circuit condition monitoring, mounted under the body of a rail vehicle. The authors used electromagnetic induction as a sensing technique, and the consequently obtained measurements were nonintrusive and galvanically isolated from the track circuit. The authors developed the physical system and accompanied it with a virtual digital twin of the rail network integrating the sensor data.

Solutions used in railway condition monitoring are also transferable to other facilities. For example, the authors of Koenig et al. [16,386] mentioned that vibration monitoring was applied to measure movement in both static structures and in rotating equipment such as wheels, and these authors developed this approach to be applied to the structure of tracks carrying high-speed rotating equipment—namely baggage cart wheels at an airport. The idea was used because unpredictable breakdowns are a serious challenge at airports, especially when wheels become blocked, and as a consequence, massive derailment of carts occurs within connection tunnels. Condition monitoring to prevent baggage cart failure in real time was applied by the authors, and was additionally enriched by autonomous systems linked to artificial intelligence and **Industry 4.0** airport logic. In Metso and Thenent [387], the authors also investigated aspects of condition monitoring in connection to maintenance 4.0 for air transport; therefore, it may be suspected that this would be a new trend in the condition monitoring of rail transport facilities.

In Xu et al. [388], the authors developed an insulation-monitoring method for railway signaling power systems (signaling power distribution networks with multiple branches), stating that intelligent condition monitoring and accurate fault location are significant for railway transport. The authors’ method allows for the continuous monitoring of the resistive earth leakage current, and indicates the locations of leakage points by tracking the rectified difference pattern. The authors’ method did not require an external power supply or signal injection. 

The term Industry 4.0 is interconnected with robotics; therefore, rail transport researchers also share an interest in this aspect of engineering.

Ahmadian et al. [389] investigated concepts to decrease the dependence on bonded insulated joints for railway track monitoring. The authors examined the use of insulated joints and discussed the utilization of track circuits according to knowledge on acoustic methods, reflectometry, and robotics. The authors considered autonomous vehicles enriched with GPS to be a form of **robotics**; therefore, this is one such early-published paper considered within this topic.

In Rowshandel et al. [246], the authors mentioned various devices that are used in the rail industry for condition monitoring—namely, inspection trains, dual-purpose road/track vehicles, and handheld trolleys utilizing ultrasonic, eddy current, and alternating current field measurements, or magnetic flux leakage **sensors**. The authors also mentioned that most devices and systems provide data on defect locations; however, the information on defect sizes is rarely indicated. Therefore, they presented a system based on **robotics**, whose purpose was the detection and characterization of rolling contact fatigue cracks in rail tracks. The authors’ system consisted of a mechanized cart, a robotic arm, a low-cost distance laser sensor, and a commercially available current field measurement system. When a defect was detected, a robotic arm located on a trolley would perform a detailed two-dimensional scan of the characterization defects. This was quite a new solution, and especially important at the time when the Industry 4.0 concept began to be broadly analyzed.

## 5. Conclusions

As mentioned in this paper’s introduction, the approaches applied to the condition monitoring of rail systems have much evolved in recent decades. Observation of these changes over the analyzed period of time has led us to define this review paper’s aim. We formulated two research questions, namely:

- RQ1: What are the current trends in condition-monitoring approaches, and how have these trends evolved over the last decades?
- RQ2: What are the future research directions and perspectives in the condition monitoring of rail transport systems?

All of the presented results, discussed both in the form of bibliometric performance analysis and systematic literature review, have headed toward finding the answers to the two mentioned research questions. Already, in terms of highlighting the subsections in this paper, the authors have identified some thematic groups in which researchers are interested, in the context of widely understood condition-monitoring applications in rail transport. These thematic groups of issues were as follows:

- various types of sensors used in different solutions applied to condition monitoring in railway transport,
- wireless and online communication, and thereby current aspects of Industry 4.0, applied to condition monitoring in railway transport.

The latter group of issues would seem especially surprising at first glance, because transport issues are seemingly not directly related to the topic of Industry 4.0 as an idea aiming at the full automation of technological and manufacturing processes. Each one of these thematic groups of issues was discussed in a chronological manner; therefore, Section 4 of this review paper—comprising Section 4.1, Section 4.2 and Section 4.3—answers the question about current trends in condition-monitoring approaches and their evolution over the past few decades. In the search for current trends, the keyword analysis that accompanied the descriptions in Section 4 was an undeniable means of finding answers, and this culminates in Table 3 and Figure 13; in Table 3, quantitative analysis of research papers related to specific keywords is indicated, while in Figure 13, quantitative and qualitative illustration of the relationships between specific keywords is presented. It is also important, for example, to analyze the countries within which the research was carried out, including in particular the cradle of rail transport—the United Kingdom—and the countries in which high-speed railway systems are developing on a large scale, as shown in the bibliometric analysis and summarized in Figure 12 and Table 2. All of the information presented in this review paper focused on the specific evolution of solutions connected to condition monitoring. At present, maintenance has been getting gradually more support from methods connected to sensor application, and the increased use of sensors has led to the currently discussed techniques focused on Industry 4.0. An enormous increase in amounts of generated data—known as big data—requires diagnostic procedures to be automated. Such a challenge induced the need for new and improved methods of autonomous interpretation of condition-monitoring data (mainly vibration methods). 

The second research question stated by the authors of this paper was “what are the future research directions and perspectives in the condition monitoring of rail transport systems?”; these directions and perspectives might be expressed by the indication of particular research agendas.

Inertial sensors such as accelerometers and gyroscopes have been applied to the condition monitoring of transportation resources since the early 1990s, Chia et al. [50]. Therefore, it can be stated that this is a much-discussed topic, and it is worth noting novel fields of exploration. The application of new concepts triggers a number of potential research agendas that may be developed in the coming years, in addition to those that are still in progress. One of these relatively novel research agendas that are connected to changes in condition monitoring results from the development of the concept of Industry 4.0. This research agenda can be expressed as a requirement for the automation of diagnostic procedures and methods of generating large amounts of data, which drives the need for more sophisticated methods of autonomous interpretation of vibration-reliant condition-monitoring data. In turn, a large amount of monitoring leads to the collaborative term of Industry 4.0 as big data. This is worth considering in accordance with the past decade’s discussion of particular aspects of big data applied in condition monitoring, such as volume, velocity, variety, veracity, and value. Furthermore, discontinuance of onboard data computing is still a challenge, and seems to be a research agenda for the next several years, especially given that the quantity of data continuously increases as an exponential or power function in relation to—for example—the number of sensors used and their measurement directions. This may be supported by the application of various methods of artificial intelligence. Another interesting research agenda highly connected to Industry 4.0 is the Internet of Things, as certain abovementioned studies suggest.

Moreover, from the viewpoint of researchers, it is significant to reduce the number of sensors as factors generating additional data in huge amounts. Some authors go even further, wondering how to analyze the condition of tracks and vehicles other than by adding more sensors to railroad systems (e.g., using passengers’ phones for this purpose is interesting). A separate issue is the analysis of different means of communication between different elements of monitoring systems, which is discussed in the second subsection of Section 4.

In general, a separate research agenda is to consider the appropriate number of sensors used for a specific need, and to determine where to locate these sensors.

At the end of this paper, it is also worth mentioning some of its limitations. One of these is the manner in which the literature references are segregated. A thematic convention was adopted in this regard, in line with the keywords, although within each topic (themes) the successive works were described in chronological order. Because of this chronological order, this may in some cases create a sense of apparent differentiation of themes, especially in cases where keywords are consolidated (which necessarily occurs in the case of most of the cited publications, as several keywords are assigned to each publication). In addition, in future analyses, it will be worth commenting on the increasing number of sensors, their diversity, and the amount of data that were acquired using these sensors, while also elaborating on the gradual abandonment of the use of sensors in track and rail vehicle condition-monitoring studies. In future considerations, it would be worth separating the topics on infrastructure and means of transport—focused directly on light rail vehicles, urban and long-distance rail transport, and high-speed trains—and additionally include a breakdown by country. 

Moreover, it is worth noting here that topics centered on the concept of Industry 4.0 have just recently entered the field of vehicle and track condition monitoring in the case of rail transport. However, “[t]he biggest challenge for condition monitoring is ensuring selection of the right technologies”, Brant and Liang [7].

## Figures and Tables

**Table 1:** The overview of definitions and approaches connected to the condition monitoring of rail transport systems. Source: own elaboration based on references directly mentioned in the table.

Definition	Year of Publication	Source
“The condition monitoring systems developed so far in rail vehicle applications are mainly based on the direct measurement of relevant signals, which are analysed using time and/or frequency domain signal processing, e.g., to find features or signatures related to particular faults [43,44]).”	2008	[45]
“(…) condition monitoring in today’s complex systems is mostly regarded as an alarm tool for maintenance [40]. Even the ISO 17359 defines, how a condition monitoring policy should look like to establish a successful maintenance strategy [41].”	2009	[42]
“Condition monitoring systems are used to collect both digital and analogue signals within a location room utilizing distributed transducers connected to either point-to-point or digital bus communication links. They often contain some kind of alarm system based around thresholding techniques, the limits being set by local maintenance personnel who have to inspect the recorded signatures daily to attempt to anticipate any failures. No diagnostic capabilities are provided. (…) Fault detection and diagnosis systems are a more advanced version of condition monitoring systems, incorporating ‘intelligent’ algorithms capable of detecting faults prior to failure, diagnosing the incipient fault and providing some indication of the criticality of the detected fault.”	2009	[39]
“Condition monitoring is necessary in order to immediately detect vehicle faults. For condition monitoring, it is necessary to detect the fault from the signals of sensors attached to the vehicles [2]. Condition monitoring can be considered to be a part of the well-established and well-developed area of Fault Detection and Isolation (or Identification) (FDI).”	2010	[38]
“Condition monitoring measures are crucial to ensure safe and cost-effective train operation in the railroad transportation industry. A well-designed monitoring system substantially reduces hardware maintenance cost and improves service quality and overall safety.”	2012	[37]
“Condition monitoring technology within the railway industry has proliferated in recent years; this is due to the continuous improvement of electronic-based systems. This has created a unique situation for implementing proactive condition monitoring technology in the railway industry. This approach will create the possibilities of identifying failing systems while the asset is in operation before they create catastrophic damage. Economically, most of these proactive products are wayside condition monitoring systems and very few sensors are few sensors are directly mounted on the vehicles.”	2012	[6]
“Condition monitoring of vehicles allows to track the development of their technical degradation, which allows to implement rational preventive and remedial activities in order to avoid unpredictable downtimes related to the damages and serious failures. The reason for continuous improvement of vehicles’ condition monitoring methods are stricter and stricter requirements concerning the reliability and safety of both all transport system and separate means of transport.”	2013	[36]
“Condition monitoring requires the gathering of data and processing of that data into useful information to support design, availability, reliability and maintenance practices.”	2014	[35]
“Condition monitoring aims to record the current (real-time) condition of a system [33].”	2014	[34]
“Condition monitoring detects and identifies deterioration in structures and infrastructure before the deterioration causes a failure or prevents rail operations. In simple condition monitoring, sensors monitor the condition of a structure or machinery. If the sensor readings reach a predetermined limit or fault condition, then an alarm is activated. However, this simplistic approach may lead to a large number of false alarms and missed failures [31]. (…) Condition monitoring can be performed continuously or periodically. Continuous monitoring should detect a problem straight away but it is often expensive; energy hungry, which is a problem for WSNs where the network components need power; and the sensor data are very noisy, which requires careful preprocessing to ensure accurate diagnostics. Periodic monitoring is cheaper, uses less energy, and allows time for data cleaning and filtering but a problem will only be diagnosed at the next processing run. (…) In basic condition monitoring, the system is only able to distinguish between normal and abnormal conditions (no fault or fault).”	2014	[32]
“Under this framework, the condition-based monitoring (CBM), as a technique to provide prognosis and diagnosis of component degradation, allows to detect the in-service failures and contributes to the decision-making for improving the system performance [19,26]. Distinguished from the conventional fault detection carried out in the depot, this technique is aiming at the online identification of the component performance on the operational conditions.”	2016	[30]
“Technologies that can perform condition monitoring and condition-based maintenance are thus of significant interest to identify and predict progressing degradation trends and optimally schedule maintenance actions to lower maintenance costs and downtime.”	2016	[29]
“Condition monitoring is referred to as a process of monitoring the condition of the objective where the relevant parameters are measured in order to determine the significant changes which is indicative of a developing fault. Considering the railway system, the objectives to be monitored could be: [v]ehicle components such as wheels, axle bearings and brake pads, [i]nfrastructure such as the track, rail beds and bridges or [p]assengers/goods within train cars.”	2017	[28]
“Condition monitoring can be considered to be a part of the well-established area of Fault Detection and Isolation (or Identification) (FDI) [25]. Condition monitoring is mainly applicable to system that deteriorate in time. The aim of condition monitoring is to detect and isolate deterioration before it causes a failure [2,26].”	2017	[27]
“Railway track and rolling stock condition monitoring is essential in ensuring the safe and efficient function of railway systems. The ability to use an instrumented revenue vehicle for the condition assessment of rail tracks is particularly significant because this capability will not require track access during inspection. This instrumented vehicle can also provide useful data for the definition of the rail track input conditions for the assessment of the stability of a rail vehicle when traversing along the track.”	2017	[24]
“Railway inspection is normally conducted periodically every year or several months. It may take too much time to rapidly detect faults in the track that may cause collapse or huge loss, as is the case in the prompt identification of rail defects. (…) Hence, condition monitoring of rail infrastructure has become important for setting proper predictive maintenances before defect and failure take place. (…) Condition monitoring can reduce maintenance and its costs by detecting the faults before they can cause damage or prevent rail operations [22].”	2018	[23]
“Condition monitoring is defined as the practice of monitoring parameters of a system or component, with the intent of getting deeper knowledge of how that system changes and deteriorates with time. If key parameters that can be linked to wear, faults and degradation can be monitored, emerging problems can quickly be found or even predicted.”	2018	[21]
“Condition monitoring is based on the regular acquisition of machine conditions or their components by measuring and analyzing physical quantities. These are compared by the system with specified conditions and serve for diagnosis decisions [14,18,19].”	2018	[20]
“Condition monitoring is a suitable way to detect impending vehicle failures and acoustically critical trams immediately but under the aspect of conservation of resources (exploitation of wear reserves of tribological components). In order to implement such concepts, the tram operator has to be able to quickly and accurately record the current technical condition of the vehicle in order to initiate appropriate counteractions and keep downtimes to a minimum.”	2019	[17]
“A better way to avoid breakdowns is a maintenance strategy that monitors and report in real-time the condition of a machine or device in use, a key principle of Industry 4.0, so its remaining life can be estimated. This is called ‘condition monitoring’ and ‘diagnostic engineering management’. Researchers have noted considerable evidence that ‘condition-based maintenance’ gives economic advantages in most industries and is the best available strategy for preventing unexpected system downtime [11,12,13,14,15].”	2019	[16]
“The condition monitoring of railway vehicles has traditionally relied on signal processing and knowledge-based techniques but, on the other hand, modelling techniques give great potentials due to the a priori knowledge included in the model.”	2019	[10]
“Modern condition monitoring systems have led fault diagnosis to enter the era of big data, which lead to the obsolescence of traditional physics-based fault diagnosis methods [8].”	2019	[9]
“Condition monitoring of railway vehicle and track dynamics are monitored via track-based systems or vehicle-based systems ([6] [– actualized by the authors]. Systems that are track-based are usually used for monitoring the condition of wheelsets, where vehicle-based systems are used to monitor the condition of the bogie, suspension and ride comfort. (…) Condition monitoring systems are made up of sensors and collective data processing hardware are software this all has to be mounted onto the desired rolling stock in the correct location to gain the desired results.”	2020, 2012	[6,7]
“(…) the condition of complex systems is typically monitored by a large number of different types of sensors, capturing e.g., temperature, pressure, flow, vibration, images or even video streams of system conditions, resulting in very heterogeneous condition monitoring data at different time scales.”	2020	[5]
“Condition Monitoring (CM) is an efficient and achievable way to ensure the reliability of suspension systems, therefore, numerous scholars have focused on developing suitable approaches to monitor the suspension systems ([2,3]).”	2020	[4]
“(…) two generally concerned questions of condition monitoring [are the subject of researchers’ interest—added by the paper’s authors]: (i) evaluating the diagnosability and isolatability of a system with existing sensor network; and (ii) finding out a quantity-optimum set of sensors for a desired diagnosability and isolatability of a system.”	2020	[1]

**Table 2:** Cooperation in publication and citation of the researchers cited in the case of the internal keywords “condition monitoring”, obtained using VOSviewer software (version 1.6.15).

Author	Number of Documents Published	Number of Citations Obtained	Total Link Strength Computed in the VOSviewer Software	List of Documents Published by a Researcher in a Particular Year
Akin E.	8	119	14	2018: [65,66,67], 2017: [68,69]), 2016: [70], 2014: [71], 2013: [72]
Alfi S.	11	67	11	2019: [73,74], 2017: [75], 2016: [30,76,77], 2014: [78], 2012: [79,80], 2011: [81], 2008: [82]
Anyakwo A.	5	22	13	2013: [83], 2012: [84,85], 2011: [86,87,88]
Asano A.	4	7	11	2016: [89,90], 2014: [91,92]
Asplund M.	5	35	8	2016: [93,94,95], 2014: [34,96]
Aydin I.	5	97	10	2018: [66], 2017: [68], 2016: [97], 2014: [71], 2013: [72]
Ball A.	8	119	15	2017: [98], 2015: [99], 2013: [83], 2012: [6,84,85,100], 2011: [86,87,88]
Bruni S.	15	284	14	2020: [101], 2019: [73,74], 2017: [75], 2016: [30,76,77,102], 2014: [78], 2012: [79,80], 2011: [81], 2008: [82], 2007: [26], 2006: [103]
Capolino G.-A.	6	138	5	2017: [104], 2011: [105], 2009: [106,107], 2008: [108,109]
Cole C.	5	20	0	2019: [47], 2018: [110,111,112], 2009: [113]
Dixon R.	9	156	17	2019: [48,114], 2013: [115], 2012: [116,117], 2011: [19], 2010: [118,119], 2008: [33]
Dollevoet R.	11	95	15	2019: [120], 2018: [121,122], 2017: [123,124], 2016: [125], 2015: [126,127,128], 2014: [129], 2008: [130]
Firlik B.	6	19	0	2020: [131], 2012: [132,133,134,135,136]
Galar D.	6	91	5	2016: [137], 2015: [138,139], 2014: [34], 2013: [140], 2012: [141]
Gao M.	5	40	11	2020: [142], 2019: [143], 2018: [144,145], 2017: [146]
Goebel K.	5	134	10	Lall et al. (2012a) [147], Lall et al. (2012b) [148], Lall et al. (2011a) [149], Lall et al. (2011b) [150], Lall et al. (2010) [151]
Goodall R.	15	814	36	2013: [115], 2012: [116], 2011: [19,117], 2010: [118,119], 2009: [152], 2008: [33], 2007 [25,153,154,155], 2006: [2,156], 2004: [157]
Goodman C.J.	5	202	16	2009: [158], 2007: [153,154,155], 2006: [159]
Gu F.	6	74	11	2017: [98], 2012: [6,85,100], 2011: [86,88]
Henao H.	5	122	5	2011: [105], 2009: [106,107], 2008: [108,109]
Ho S.L.	5	64	0	2012: [37], 2008: [160,161], 2006: [162,163]
Huang Z.	5	22	6	2017: [164,165], 2016: [166,167], 2014: [168]
Kaewunruen S.	7	44	3	2019: [169,170], 2018) [23,171], 2017: [164,172,173]
Karakose M.	10	94	17	2020: [174], 2018: [65,66,67,175], 2017: [68,69], 2016: [70,97], 2014: [71]
Kostrzewski M.	5	52	0	2018: [176,177], 2017: [178], 2014: [179], 2012: [180]
Kumar U.	6	14	5	2016: [137,181], 2014: [182], 2012: [141,183], 2010: [184]
Lall P.	5	134	10	2012: [147,148], 2011: [149,150], 2010: [151]
Li P.	5	411	16	2007: [153,154,155], 2006: [159], Li et al. (2004) [157]
Li Y.	8	37	5	2020: [185,186], 2019: [187,188], 2018: [144], 2017: [104], 2016: [12], 2011: [189]
Li Z.	14	152	14	2020: [190], 2019: [187,191], 2018: [56,121], 2017: [123,124], 2016: [125], 2015: [126,127,128], 2014: [129,192], 2008: [130]
Liu X.	6	9	5	2020: [193], 2019: [194,195], 2018: [196], 2016: [197], 2014: [198]
Lowe R.	5	134	10	2012: [147,148], 2011: [149,150], 2010: [151]
Markine V.L.	6	9	5	2020: [193], 2019: [195], 2018: [196,197], 2014: [198,199]
Matsumoto A.	9	48	23	2020: [200], 2018: [201,202], 2014: [92], 2012: [203], 2011: [204], 2008: [205,206], 2006: [207]
Mei T.X.	6	347	9	2014: [208], 2011: [19], 2009: [45], 2008: [209,210], 2007: [26]
Mori H.	6	38	15	2016: [89,90], 2014: [91,92], 2011: [38,204]
Márquez F.P.G.	13	759	10	2017: [211], 2016: [212,213], 2015: [214,215], 2012: [216,217], 2011: [218], 2010: [39,219], 2008: [220], 2007: [221,222], 2003: [223]
Naganuma Y.	5	50	6	2014: [224,225], 2013: [226], 2011: [204], 2008: [227]
N*úñe*z A.	9	63	12	2019: [120], 2018: [122], 2017: [123,124], 2016: [125], 2015: [126,127,128], 2014: [129]
Ohno H.	5	30	15	2020: [200], 2018: [201,202], 2012: [203], 2006: [207]
Palo M.	9	52	9	2016: [93,94,95], 2014: [34,96], 2013: [228], 2012: [141,183], 2012: [229]
Papaelias M.	12	605	14	2020: [230], 2019: [170], 2017: [164,165,211,231], 2016: [166,213], 2014: [167,168], 2012: [216], 2011: [218]
Pislaru C.	7	87	15	2014: [232], 2013: [83], 2012: [6,84,85], 2011: [86,88]
Rantatalo M.	6	25	7	2019: [233], 2016: [93,94,95], 2014: [96,182]
Roberts C.	34	748	53	2020: [230,234], 2019: [235,236], 2017: [237], 2015: [214,238,239], 2014: [240,241,242,243], 2011: [19,244,245,246], 2010: [39,219,247], 2009: [152,158,248], 2008: [249,250,251], 2007: [153,154,155,222], 2006: [2,31,156,159,252]
Sato Y.	5	30	15	2020: [200], 2018: [201,202], 2012: [203], 2006: [207]
Stewart E.	11	70	16	2020: [230,234], 2019: [48,236], 2015: [238,253,254], 2011: [19,244,249,250]
Suda Y.	6	0	2	2019: [255], 2018: [256], 2016: [257,258], 2014: [259], 2012: [260]
Tanimoto M.	8	30	17	2020: [200], 2019: [261], 2018: [201,202], 2016: [257,258], 2012: [203], 2006: [207]
Tobias A.M.	6	578	10	2015: [215], 2012: [216], 2010: [39,219], 2009: [158], 2008: [251]
Tsunashima H.	12	274	23	2016: [89,90], 2014: [91,92,224,225], 2013: [226], 2011: [204], 2010: [262], 2008: [205,206], 2007: [26]
Wang P.	5	40	11	2020: [142], 2019: [143], 2018: [144,145], 2017: [146]
Wang Y.	6	40	11	2020: [142,263], 2019: [143], 2018: [144,145], 2017: [146]
Ward C.P.	6	92	14	2013: [115], 2012: [116], 2011: [19,117], 2010: [118,119]
Weston P.	20	522	42	2020: [230,234], 2018: [264], 2015: [238,239], 2014: [24,242,243], 2011: [19,244,245], 2008: [249,250], 2007: [153,154,155,222], 2006: [156,159,252]
Yaman O.	6	41	11	2020: [174], 2018: [65,66,67,175], 2017: [69]
Zhang W.	5	26	1	2020: [265], 2019: [266], 2018: [267], 2017: [237], 2016: [12]

**Table 3:** Countries’ specific cooperation in the research on “condition monitoring”, obtained using VOSviewer software (version 1.6.15).

Country	Number of Documents Published	Number of Citations Obtained	Total Link Strength Computed in the VOSviewer Software	Country	Number of Documents Published	Number of Citations Obtained	Total Link Strength Computed in the VOSviewer Software
United Kingdom	164	2776	54	Finland	4	257	0
China	71	447	35	Norway	4	13	2
Japan	45	387	8	Portugal	4	53	7
Italy	42	981	15	South Korea	4	199	0
United States	41	755	10	Ireland	3	10	5
Netherlands	33	171	11	Latvia	3	15	5
Sweden	32	258	11	Switzerland	3	6	5
Spain	25	938	20	Greece	2	1	0
India	22	97	6	Hungary	2	1	0
Poland	20	151	0	Iran	2	14	0
Australia	19	225	11	Mexico	2	16	1
France	18	192	8	Romania	2	0	0
Turkey	14	141	2	Tunisia	2	18	2
Hong Kong	13	153	7	United Arab Emirates	2	2	0
Germany	12	22	0	Indonesia	1	2	1
Canada	8	4	4	Malaysia	1	1	1
Czech Republic	7	43	2	Myanmar	1	0	1
Russian Federation	7	58	3	Nigeria	1	3	0
Singapore	7	21	0	Qatar	1	1	3
Austria	6	8	0	Saudi Arabia	1	1	1
Denmark	6	20	2	Serbia	1	2	1
South Africa	5	146	2	Taiwan	1	1	0
Belgium	4	64	6	Uganda	1	1	0

**Table 4:** The list of review papers’ specific keywords.

Keyword	Occurrences	Total Link Strength	Appearance of a Particular Keyword in the Following Publications
Various types of sensors			
3D acceleration sensor	1	0	2014: [198]
acceleration sensors	1	0	2018: [268]
accelerometer	4	3	2020: [263], 2014: [275], 2012: [270], 2011: [271]
accelerometers	24	13	2020: [142,263,288], 2019: [50,281,284,289], 2018: [110,278,279,282], 2017: [146,280,390], 2016: [166,276], 2014: [91,242], 2013: [274], 2010: [118,119], 2007: [153,154], 2006: [156], 2001: [269]
acoustic emission sensors	4	1	2017: [165], 2016: [166], 2014: [168], 1995: [286]
angular rate sensor	1	0	2014: [259]
contrast sensor	1	0	2011: [291]
eddy current sensor	1	0	2019: [233]
electric sensing devices	5	6	2018: [297], 2017: [294], 2014: [293,296], 2012: [37]
fiber-optic sensors	12	9	2020: [190,308], 2019: [304,306], 2017: [294,295], 2016: [298], 2015: [303], 2012: [37,301], 2006: [299], 2003: [353]
fiber optics	6	4	2019: [306,358], 2017: [295], 2016: [359], 2006: [103,299]
fiber Bragg grating sensors	5	3	2016: [302], 2012: [300,301], 2008: [160,161]
gyroscopes	11	7	2020: [263], 2019: [50,289], 2014: [91,242], 2013: [274], 2008: [205,206], 2007: [153,154], 2006: [156]
heterogeneous sensors	1	0	2016: [321]
inertial sensor	9	2	2020: [322], 2019) [50,374], 2017: [350], 2012: [116], 2011: [19,117,245], 2009: [45]
inertial measurement sensors	1	0	2019: [289]
infrared proximity sensor	1	0	2013: [292]
laser scanning	4	0	2016: [93,94], 2014: [324,327]
laser displacement sensors	1	0	2019: [326]
laser sensor	1	0	2011: [245]
MEMS sensors	1	0	2019: [284], 2017: [280]
multiple sensors	1	0	2018: [328]
onboard sensors	1	0	2020: [349]
optical fibers	6	8	2019: [304,305,358], 2017: [294], 2016: [298,302]
optical sensors	3	3	2018: [360], 2016: [354], 2007: [153]
optical fiber Bragg grating strain sensor	1	0	2012: [300]
optoelectronic and photonic sensors	1	0	2018: [360]
piezoelectric sensors	1	0	2018: [297]
pressure and temperature sensors	1	0	2017: [329]
rechargeable sensors	1	0	2019: [143]
remote sensors	2	0	2017: [49]
sensor	7	2	2020: [307,338,339,340,363,371], 2019: [50,287]
sensors	26	17	2020: [371], 2019: [17,47,50], 2018: [357], 2014: [334], 2013: [272,273,292,333], 2012: [6,132,148,203,332], 2011: [19,38,105,117,245,246,291], 2010: [330], 2009: [106,107], 2008: [108,109], 2007: [26,53,153], 2006: [159,299], 2002: [52,331], 1995: [286]
sensor data fusion	3	2	2018: [323,337], 2017: [335]
sensor fusion	4	4	2020: [263], 2018: [323,336,337]
sensor networks	5	7	2018: [267], 2017: [49,293], 2014: [294], 2011: [245]
sensor nodes	13	26	2020: [142,363], 2019: [17,47,143], 2018: [144,145,146,361], 2016: [344,354,355], 2014: [293]
strain gauge sensor	1	0	2012: [100]
strain sensors	3	3	2016: [315], 2012: [300], 2012: [100]
vibration estimating sensors	1	0	2018: [283]
wired sensor	1	0	2018: [23]
vibration sensor	2	4	2020: [340], 2018: [323]
vibration sensors	4	7	2020: [340], 2019: [17], 2018: [264], 2013: [272,273]
Wireless and online communication			
Bluetooth communication	2	0	2012: [341], 2011: [342]
GPS	19	3	2020: [322,351], 2019: [347], 2018: [112,346], 2017: [350], 2016: [89,90,344,345,352], 2014: [91,92,242], 2013: [273], 2011: [204], 2008: [205,206,343]
map-matching algorithm	4	3	2014: [91,92], 2011: [204], 2008: [205],
smartphones	4	0	2020: [349,351], 2017: [350], 2015: [348]
wireless communications	2	7	2020: [362], 2018: [145], 2015: [32]
wireless networks	2	4	2016: [354], 2010: [379]
wireless sensor network	3	9	2019: [17,47,143]
wireless sensor network (WSN)	2	2	2018: [23] 2018: [283]
wireless sensor networks (WSNs)	1	5	2015: [32]
wireless sensor networks	15	28	2019: [17,47,143], 2018: [23,144,145,283,357,361,380], 2016: [354,355], 2015: [32], 2014: [356]
wireless sensor node	4	8	2020: [363], 2019: [143], 2018: [144,145]
Current aspects of industry 4.0			
artificial intelligence	12	7	2020: [5,369,371], 2019: [368], 2018: [65,112,175] 2015: [127,367], 2013: [365], 2012: [364], 2006: [290]
big data	10	8	2020: [378], 2019: [9,255,373,374,375,376]], 2018: [256,257,258], 2015: [126]
Industry 4.0	5	0	2020: [174,372,387], 2019: [16,368]
intelligent condition monitoring	5	0	2020: [381], 2018: [122,388], 2015: [214], 2013: [382], 2006: [252]
intelligent systems	5	1	2020: [46], 2019: [194], 2014: [129,383], 2013: [83]
Internet of Things	7-	12	2020: [378,381], 2019: [374], 2018: [65,145,268,380]
Internet of Things (IoT)	3	6	2020: [378,381], 2018: [65]
robotics	3	1	2016: [321], 2011: [246], 2005: [389]

## Data Availability

Not applicable.
